# impMKT: the imputed McDonald and Kreitman test, a straightforward correction that significantly increases the evidence of positive selection of the McDonald and Kreitman test at the gene level

**DOI:** 10.1093/g3journal/jkac206

**Published:** 2022-08-17

**Authors:** Jesús Murga-Moreno, Marta Coronado-Zamora, Sònia Casillas, Antonio Barbadilla

**Affiliations:** Institute of Biotechnology and Biomedicine, Universitat Autònoma de Barcelona, Barcelona 08193, Spain; Department of Genetics and Microbiology, Universitat Autònoma de Barcelona, Barcelona 08193, Spain; Institute of Biotechnology and Biomedicine, Universitat Autònoma de Barcelona, Barcelona 08193, Spain; Department of Genetics and Microbiology, Universitat Autònoma de Barcelona, Barcelona 08193, Spain; Institute of Biotechnology and Biomedicine, Universitat Autònoma de Barcelona, Barcelona 08193, Spain; Department of Genetics and Microbiology, Universitat Autònoma de Barcelona, Barcelona 08193, Spain; Institute of Biotechnology and Biomedicine, Universitat Autònoma de Barcelona, Barcelona 08193, Spain; Department of Genetics and Microbiology, Universitat Autònoma de Barcelona, Barcelona 08193, Spain

**Keywords:** natural selection, McDonald and Kreitman test, nucleotide variation, positive selection, protein-coding genes

## Abstract

The McDonald and Kreitman test is one of the most powerful and widely used methods to detect and quantify recurrent natural selection in DNA sequence data. One of its main limitations is the underestimation of positive selection due to the presence of slightly deleterious variants segregating at low frequencies. Although several approaches have been developed to overcome this limitation, most of them work on gene pooled analyses. Here, we present the imputed McDonald and Kreitman test (impMKT), a new straightforward approach for the detection of positive selection and other selection components of the distribution of fitness effects at the gene level. We compare imputed McDonald and Kreitman test with other widely used McDonald and Kreitman test approaches considering both simulated and empirical data. By applying imputed McDonald and Kreitman test to humans and *Drosophila* data at the gene level, we substantially increase the statistical evidence of positive selection with respect to previous approaches (e.g. by 50% and 157% compared with the McDonald and Kreitman test in *Drosophila* and humans, respectively). Finally, we review the minimum number of genes required to obtain a reliable estimation of the proportion of adaptive substitution (*α*) in gene pooled analyses by using the imputed McDonald and Kreitman test compared with other McDonald and Kreitman test implementations. Because of its simplicity and increased power to detect recurrent positive selection on genes, we propose the imputed McDonald and Kreitman test as the first straightforward approach for testing specific evolutionary hypotheses at the gene level. The software implementation and population genomics data are available at the web-server imkt.uab.cat.

## Introduction

Natural selection leaves characteristic footprints at the patterns of genetic variation. Since the advent of next-generation sequencing, numerous statistical methods have been proposed to analyze genomic data (Casillas and Barbadilla 2017), allowing the detection and quantification of molecular adaptation at different temporal scales. The McDonald and Kreitman test (MKT) (McDonald and Kreitman 1991) is one of the most powerful and robust methods to detect the action of recurrent natural selection at the DNA level. Unlike the *ω* ratio (Kimura 1977), which compares the number of synonymous (*D_S_*) and nonsynonymous (*D_N_*) divergent sites, the MKT combines both divergence (*D_S_*, *D_N_*) and polymorphism (*P_S_*, *P_N_*) data. Polymorphic data allows taking into account purifying selection on divergent nonsynonymous sites, significantly increasing the power of detecting recurrent positive selection.

The null model of the original MKT approach is the neutral theory (Kimura 1968, 1977; Ohta 1973). It assumes that positively selected (adaptive) mutations are rare, and thus not easily observable when polymorphic sequences are sampled at a given time *t*, thus contributing almost exclusively to divergence (and not to polymorphism). Therefore, an excess of the divergence ratio relative to the polymorphism ratio is the signal of positive selection acting on nonsynonymous sites ($D_{N}/D_{S}$ > $P_{N}/P_{S}$). Temporally, the MKT covers the evolutionary period spanning from the present to the time back to divergence between the target and the outgroup species, and it allows the estimation of the fraction of adaptive nonsynonymous substitutions (*α*) (Charlesworth 1994; Smith and Eyre-Walker 2002). Nonetheless, the MKT, as originally formulated, has multiple drawbacks that could bias the estimation of *α*. First, the MKT assumes strict neutrality on segregating (polymorphic) sites. However, several studies in multiple species have shown an excess of low-frequency variants (Smith and Eyre-Walker 2002; Messer and Petrov 2013; Galtier 2016). These variants are attributed to slightly deleterious mutations (SDM), which will not usually reach fixation, contributing more to polymorphism than divergence. SDM reduce the MKT statistical power and underestimate *α* (Eyre-Walker and Keightley 2009). Second, MKT assumes that the neutral mutation rate is constant over time and so is the selective constraint. However, the nearly neutral mutation rate depends on the effective population size (*N_e_*) (Balloux and Lehmann 2012; Lanfear *et al.* 2014; Rousselle *et al.* 2018; Galtier and Rousselle 2020) and, therefore, changes in population size can affect the MKT considerably. SDM get fixed at higher rates in populations with past smaller sizes, contributing to divergence and leading to an overestimation of *α* (Eyre-Walker and Keightley 2009). Besides, recent evidence shows that weakly advantageous mutations can also be segregating within populations (Galtier 2016; Tataru *et al.* 2017; Uricchio *et al.* 2019). The presence of this positively selected polymorphism, like SDM, can mask the effect of adaptive selection, since it counteracts the excess of the divergence ratio relative to the polymorphism tested by the MKT.

Over the last decades, several modifications in the original MKT have been proposed to account for the potential biases in the estimation of *α* (Templeton 1996; Fay *et al.* 2001; Eyre-Walker and Keightley 2009; Mackay *et al.* 2012; Messer and Petrov 2013; Galtier 2016). Most of these extensions deal with the presence of SDM. Although other forces affect the site frequency spectrum (SFS) of segregating variants, such as recombination, demography, ancestral population sizes, or weak positive selection, several studies have pointed out the relevance of SDM (Eyre-Walker *et al.* 2006; Eyre-Walker and Keightley 2009). SDM distort the nonsynonymous SFS and have been repeatedly shown to be a main factor biasing *α* downwards (Charlesworth and Eyre-Walker 2008; Eyre-Walker and Keightley 2009; Fay *et al.* 2001; Galtier 2016).

New model-based approaches for the estimation of *α* have benefited from the increasing number of genomics data sets available, which allow dealing, implicitly or explicitly, with the underlying distribution of fitness effects (DFE) of new mutations, including the presence of SDM or controlling for correlated genomic features (Eyre-Walker and Keightley 2009; Messer and Petrov 2013; Galtier 2016; Tataru *et al.* 2017; Uricchio *et al.* 2019; Huang 2021). However, these advanced methodologies need extensive data sets to fit complex parametric evolutionary models by applying maximum likelihood (ML) inference, exponential fitting or generalized linear models and they work properly for genome-wide analyses or on large pools of genes. In contrast, these methodologies are rarely applicable over specific genes to test particular evolutionary hypotheses, as the original MKT does (McDonald and Kreitman 1991).

While more and more genome-wide analyses of evolution of protein coding genes have been carried out through these MKT extensions, the simple G-test or the independence chi-square test of the original MKT (McDonald and Kreitman 1991) is currently almost deprecated. Most MKT heuristic alternatives exclude all variants below a frequency threshold for the minor frequency allele (MAF) (Templeton 1996; Akashi 1999; Fay *et al.* 2001). Since the MAF distribution resembles an exponential one, dropping these data inevitably leads to the loss of most of the polymorphic information, consequently performing very poorly on gene-by-gene testing.

Here, we present the imputed MKT (impMKT), a modification of the Fay, Waycoff, and Wu MKT approach (fwwMKT) (Fay *et al.* 2001) to improve gene-by-gene analyses. We propose a methodology that imputes the proportion of SDM at the SFS rather than removing all variants below a frequency threshold. The impMKT maximizes the information to test the excess of divergence ratio relative to polymorphism at the gene level. We compare our imputation method to previous and recent MKT approaches, using simulated data to test its accuracy and efficiency. Moreover, we test the impMKT on the human African lineage samples of the 1000 Genome Project (1000GP) (Auton *et al.* 2015) and the Zambian population of the *Drosophila* Genome Nexus (DGN) (Lack *et al.* 2016). impMKT considerably increases the number of statistically significant genes under positive selection in Drosophila and humans, respectively, compared to other MKT approaches. Despite the limitations of heuristic MKT and MKT-derived methods, the impMKT has the advantages of simplicity, intuitiveness, ease of use, and increased statistical power to test recurrent positive selection on genes; thus, it can be used as a first straightforward approach for testing specific evolutionary hypotheses at the gene level.

## Materials and methods

### Simulated data

We used SLiM 3 (Haller and Messer 2019) to test the accuracy and performance of the impMKT compared to other MKT approaches on simulated data. We tested 15 different genetic scenarios following the procedure proposed by Campos and Charlesworth (2019) and Booker (2020).

We simulated the evolution of a population of 10,000 diploid individuals for 220,000 generations while setting a uniform population-scaled mutation and recombination rates of $4N_{e}r=4N_{e}\mu =0.001$. To improve performance, we rescaled by a factor of 10 and substitutions were recorded $14N_{e}$ generations after burn-in following Booker (2020). Each simulation contained 7 genes spaced by 8,100 bp neutral intergenic regions. For each gene, we simulated 5 exons of 300 bp separated by 100 bp neutrally evolving introns. We assumed a proportion of 0.25 and 0.75 for synonymous and nonsynonymous alleles, respectively. Deleterious alleles were modeled following a Gamma distribution, whereas beneficial alleles were modeled following a point-mass distribution. We assumed that the Gamma distribution of deleterious alleles followed a shape parameter (*β*) of 0.3, and population-scaled selection coefficients of $2N_{e}s_{-}=2000$. For beneficial alleles, we assumed a population-scaled selection coefficients $2N_{e}s_{+}=250$. We solved the analytical estimations described in Uricchio *et al.* (2019) using the corresponding software (https://github.com/uricchio/mktest) to input the fixation probabilities of strong and weakly beneficial alleles. We considered a total adaptation rate of α=0.4 while setting as 50% the proportion of adaptation due to weakly beneficial alleles ($\alpha_{W}=\alpha \cdot 0.5$). We used the estimated fixation probabilities to define the relative proportion of strong beneficial, weak beneficial, and deleterious alleles as *p_s_*, *p_w_*, and $0.75-p_{s}-p_{w}$, respectively, in our model.

We performed 2,000 replicas, totalizing 14,000 simulated genes (2,000 replicas × 7 genes), sampling 20 individuals. Besides, 7 parameters were modified to test for multiple scenarios (see Table 1). Each scenario independently replaces a genetic feature to identify limitations and advantages of the method regarding the underlying DFE, the global adaptation rate, or the number of polymorphic sites.

**Table 1.** SLiM simulated parameters.

Simulations	*N_e_*	Samples	$2N_{e}s_{-}$	$2N_{e}s_{+}$	*β*	*p_a_*	*ρ*	*θ*	Genes
Baseline	10,000	20	−2,000	250	0.3	0.00021	0.001	0.001	14,000
$2N_{e}s+=500$	10,000	20	−2,000	500	0.3	0.00012	0.001	0.001	14,000
$2N_{e}s+=100$	10,000	20	−2,000	100	0.3	0.00048	0.001	0.001	14,000
$2N_{e}s-=1000$	10,000	20	−1,000	250	0.3	0.00021	0.001	0.001	14,000
$2N_{e}s-=500$	10,000	20	−500	250	0.3	0.00021	0.001	0.001	14,000
β=0.1	10,000	20	−2,000	250	0.1	0.00115	0.001	0.001	14,000
β=0.2	10,000	20	−2,000	250	0.2	0.00048	0.001	0.001	14,000
28,000 genes	10,000	20	−2,000	250	0.3	0.00021	0.001	0.001	28,000
2,000 genes	1,000	20	−2,000	250	0.3	0.00021	0.001	0.001	2,000
ρ=0.01	10,000	20	−2,000	250	0.3	0.00021	0.01	0.001	14,000
ρ=0.0001	10,000	20	−2,000	250	0.3	0.00021	0.0001	0.001	14,000
θ=0.01	10,000	20	−2,000	250	0.3	0.00021	0.001	0.01	14,000
θ=0.0001	10,000	20	−2,000	250	0.3	0.00021	0.001	0.0001	14,000
α=0.1	10,000	20	−2,000	250	0.3	0.000036	0.001	0.001	14,000
α=0.1	10,000	20	−2,000	250	0.3	0.00075	0.001	0.001	14,000

*N_e_*: effective population size; $2N_{e}s$: population-scaled selection coefficient; *β*: shape parameter of the Gamma distribution; *p_a_*: relative proportion of advantageous mutations; *ρ*: population-scaled recombination rate; *θ*: population-scaled mutation rate; and *α*: proportion of adaptive mutation.

### *Drosophila* *melanogaster* and human data

We followed the pipeline described at Murga-Moreno *et al.* (2019) to retrieve polymorphic and divergence genome data from *D. melanogaster* and the human lineage.

In brief, for *D. melanogaster* we retrieved polymorphic and divergence data from the DGN data, using the genome sequence of *Drosophila simulans* as outgroup (release 2) (Lack *et al.* 2016). Specifically, we subset data from 13,753 protein-coding genes from the Zambian population (197 individuals). We binned the output SFS considering a sample of 20 individuals. The ancestral state of each segregating site was inferred from the sequence comparison with the outgroup species *D. simulans*. The *D. melanogaster* genome reference sequence and annotations correspond to the 5.57 FlyBase release. Gene-associated recombination rate estimates at 100-kb nonoverlapping windows were retrieved from Comeron *et al.* (2012).

For the human lineage, we retrieved polymorphic data and ancestral states for all African populations of the 1000GP Phase III (Auton *et al.* 2015). We used chimpanzee (*Pan troglodytes*) as the outgroup species to compute human divergence metrics. We downloaded hg19-panTro4 alignment from PopHuman (Casillas *et al.* 2018). Annotations retrieved from GENCODE (release 27) (Derrien *et al.* 2012) were used to assess the functional class of each genomic position. Recombination rate values associated with each protein-coding gene were obtained from Bhérer *et al.* (2017) and correspond to the sex-average estimates. We retrieved polymorphic and divergence data from 20,643 protein-coding genes. We binned the output SFS considering a sample of 20 individuals.

### MKT approaches

To test the performance and accuracy of the impMKT, we compared it against 4 already published heuristic MKT methods:

- the original MKT (McDonald and Kreitman 1991);
- the Fay, Wickoff and Wu correction (fwwMKT) (Fay *et al.* 2001) (see next section);
- the extended MKT (eMKT) (Mackay *et al.* 2012) where *P_N_*, the count of segregating sites in the nonsynonymous class is decomposed in neutral and weakly deleterious variants. Deleterious variants are assumed be only below a frequency threshold and the remaining neutral fraction is estimated from the synonymous class (*P_S_*). For a 15% threshold, the estimators are as follows:
  $$\begin{array}{l} {\hat{f}}_{\text{neutral}\;j<15\%}=\frac{P_{S(j<15\%)}}{P_{S}}\\ P_{N_{\text{neutral}\;j<15\%}}=P_{N}\times f_{\text{neutral}\;j<15\%}\\ P_{N_{\text{neutral}}}=P_{N_{\text{neutral}\;j<15\%}}+P_{N\;j\geq 15\%} \end{array}$$
  The estimated $P_{N_{\text{neutral}}}$ values can be used to perform both MKT and *α* estimations.
- the asymptotic MKT (aMKT) (Messer and Petrov 2013), which defines *α* as a function that depends on the SFS of alleles. Hence, *α* is estimated in different frequency intervals. Given the frequency spectrum distribution in the frequency interval [0,1], the estimate of *α_x_* results in an exponential function of the form $\alpha_{\left(x\right)}=a+b\cdot e^{-cx}$. The best fit of the exponential at *x* = 1 eliminates the effect of SDM. We followed Haller and Messer (2017) to choose the cutoffs for the aMKT estimations.

In addition, we included the Grapes software (Galtier 2016), an ML method fitting the DFE. We ran Grapes using the Gamma-zero DFE distribution and estimated *α* for 100 bootstrap datasets. In addition, when incorporating weak adaptation in the simulations, a gamma-exponential was run. We measured *α* confidence interval (CI) through the boundaries for *α* estimation in Grapes using α_down and α_up parameters independently for each bootstrapped dataset (Galtier 2016).

aMKT and Grapes (as well other DFE-related methods) are commonly used to estimate *α* using a large pool of genes or genome-wide data (Messer and Petrov 2013; Rousselle *et al.* 2019). Both methodologies have been previously shown to perform the most accurate estimations in the presence of SDM and demography events (Eyre-Walker and Keightley 2009; Messer and Petrov 2013). Since impMKT is specially designed to perform gene-by-gene analyses, we tried to determine in which cases the amount of data was large enough to perform estimations using aMKT and Grapes compared to impMKT.

## Results

### impMKT

Our main goal is to devise a derived MKT approach that enhances the power to detect selection at the gene level. To do this, we modified the approach proposed by Fay *et al.* (2001) (fwwMKT), which removes all nonsynonymous (*P_N_*) and synonymous (*P_S_*) polymorphic sites below a derived allele frequency cutoff *j*, assuming that SDM segregate at low frequencies. Removing variants below a cutoff, typically 5% or 15% (Fay *et al.* 2001; Mackay *et al.* 2012), implies losing a considerable amount of data. Consider the example of Nielsen and Slatkin (2013) for the standard neutral coalescence model: a 15% cutoff implies up to 44% of excluded variants of the expected SFS for a sample of *n *=* *10 haploid individuals. We observed the same trend considering the *D. melanogaster* and human data, for which considering a virtual gene containing the mean polymorphic level a 15% cutoff implies up to 80% of excluded variants of the expected SFS for *D. melanogaster* and up to 90% in humans. This amount of data exclusion may make the computation of the MKT impracticable, especially in species with low levels of polymorphism.

Here, we propose a new MKT approach that modifies the fwwMKT to impute the actual number of SDM (*P_wd_*) segregating within *P_N_*. The resulting approach, impMKT, just removes the imputed number of SDM instead of all polymorphism segregating below a given threshold as fwwMKT does, thus retaining a larger fraction of the data and increasing the power to detect positive selection.

**Fig. 1.**
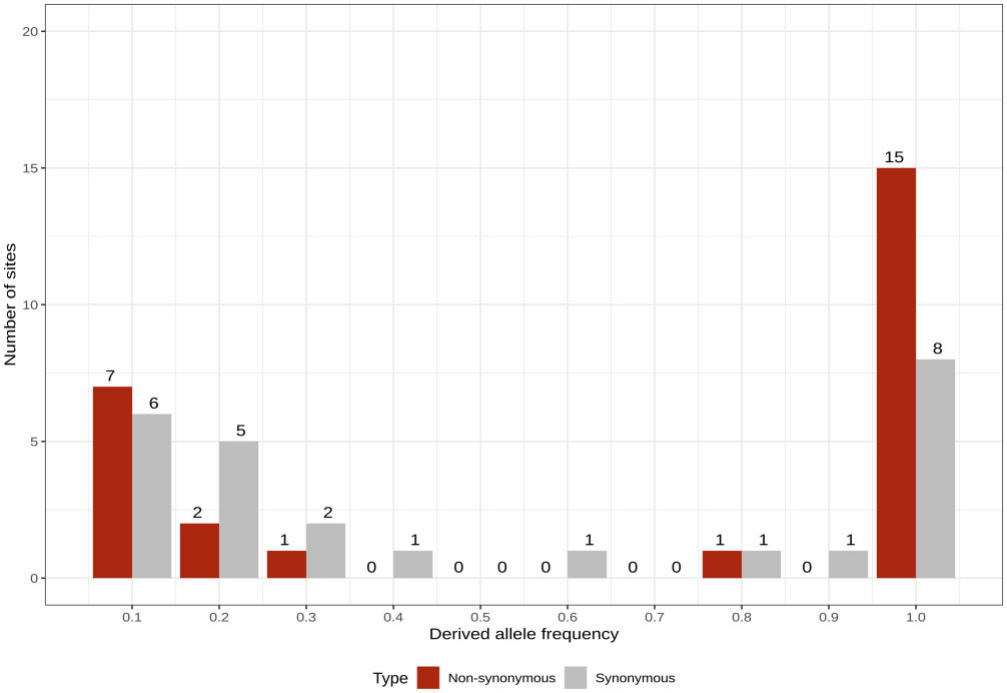
Hypothetical SFS and fixed differences from Hahn (2018).

Consider the SFS and fixed differences of a hypothetical gene as illustrated by Hahn (2018) (Fig. 1). Table 2 shows the 2 × 2 contingency tables to perform the original MKT, fwwMKT and impMKT. Charlesworth and Eyre-Walker (2008) investigated how the removal of low-frequency polymorphism affects the estimation of different MKT approaches depending on the continuous function defining the DFE for different nonarbitrary cutoffs. To develop the impMKT, we followed Charlesworth and Eyre-Walker (2008) results, which show that any derived allele frequency cutoff j>15% is a near-optimal solution to the problem of SDM segregating at the SFS (Charlesworth and Eyre-Walker 2008).

**Table 2.** Contingency tables.

	Polymorphism	Divergence
(A) Definition of the MKT 2 × 2 contingency table		
Nonsynonymous	$P_{\text{Neutral}}=P_{N}-P_{wd}$	*P_S_*
Synonymous	*D_N_*	*D_S_*
(B) Example of MKT 2 × 2 contingency table. Including all polymorphic sites		
Nonsynonymous	11	15
Synonymous	17	8
2 × 2 Fisher exact test; *P*-value = 0.093		
(C) Example of fwwMKT 2 × 2 contingency table. Removing all polymorphic sites with a derived allele frequency below 15%		
Nonsynonymous	11 − 7 = 4	15
Synonymous	17 − 6 = 11	8
2 × 2 Fisher exact test; *P*-value = 0.045		
(D) Example of impMKT 2 × 2 contingency table. Removing the expected SDM with a derived allele frequency below 15% [see Equation (2)]		
Nonsynonymous	11 − 5 = 6	15
Synonymous	17	8
2 × 2 Fisher exact test; *P*-value = 0.017		

Consequently, considering that SDM are the main force biasing *α* downward and assuming that SDM do not segregate at frequencies above 15% ($P_{wd}\to 0$), we impute the actual proportion of SDM (*P_wd_*) segregating below the frequency cutoff by considering that the expected neutral polymorphism nonsynonymous/synonymous ratio is $P_{N_{\left(j>15\%\right)}}/P_{S_{\left(j>15\%\right)}}$. This ratio can be used to infer the number of SDM in our data set (*P_wd_*). If $P_{wd}\neq 0$ bellow j<15%, then $P_{N_{\left(j<15\%\right)}}/P_{S_{\left(j<15\%\right)}}$ exceeds the expected polymorphic ratio because $P_{N_{\left(j<15\%\right)}}$ includes *P_wd_*. That is, $P_{N_{\left(j<15\%\right)}}=P_{{\text{neut}}_{\left(j<15\%\right)}}+P_{wd_{\left(j<15\%\right)}}$, where $P_{{\text{neut}}_{\left(j<15\%\right)}}$ refers to the number of nonsynonymous segregating sites that are effectively neutral. Accordingly, we can estimate (impute) *P_wd_* from expression

(1) $$\frac{P_{N(j<15\%)}-P_{wd(j<15\%)}}{P_{S(j<15\%)}}=\frac{P_{N(j>15\%)}}{P_{S(j>15\%)}}$$

rearranging we have

(2) $$P_{wd}\approx P_{wd(j<15\%)}=P_{N(j<15\%)}-\frac{P_{N(j>15\%)}\cdot P_{S(j<15\%)}}{P_{S(j>15\%)}}$$

Considering our example in Table 2, *P_wd_* is

(3) $$P_{wd}=7-\frac{4\cdot 6}{11}\approx 5$$

And, thus, 5 is the number of sites removed from the nonsynonymous polymorphism counts (see Table 2D).

As can be seen in Table 2C, the approach proposed by Fay *et al.* (2001) shows that removing all low-frequency polymorphisms below a given threshold *j* significantly increases the power of detection of positive selection by conducting a 2 × 2 test. Thus, testing for the ratio of replacement on fwwMKT 2 × 2 contingency table through a Fisher exact test decreases the *P*-value significance from 0.093 to 0.045 in our example. Nonetheless, it implies a reduction of 46% of the analyzed data, reducing *P_N_* from 11 to 4 and *P_S_* from 17 to 11, respectively. In comparison, by simply removing the expected number of SDM (*P_wd_*), we reduced the data loss to only 15%, while decreasing the *P*-value from 0.093 to 0.017 (see Table 2D).

Therefore, the impMKT allows maximizing gene-by-gene analyses where information is limited to a small number of polymorphic sites. Note that in cases where data is highly constrained, SDM would not rise in frequency, or their presence would be negligible, and the impMKT will be penalized since the imputation removes data from the 2 × 2 contingency table. In such cases, impMKT should be avoided, and the method developed by Eilertson *et al.* (2012) shows better performance than the original MKT for several genetic scenarios.

In addition, we can correct *α*, the proportion of adaptive substitutions, by removing the expected proportion of SDM (*P_wd_*) with the expression

(4) $$\alpha_{\text{imputed}}=1-\left(\frac{P_{N}-P_{wd}}{P_{S}}\times \frac{D_{N}}{D_{S}}\right)$$

#### Other selection regimes

The SDM imputation can be used to estimate other selective components shaping the DFE. Let consider the model proposed by Eyre-Walker and Keightley (2009) and nearly neutral theory (Ohta 1973), where selected segregating alleles are drawn from a continuous Gamma distribution and categorized as strongly deleterious, slightly deleterious and effectively neutral mutations. Analogous to Mackay *et al.* (2012), we define the statistics *d*, *d_w_*, and *d*_0_, which measure the different types of purifying selection, both at genome-wide and gene levels. These measures are like heuristic estimates of the DFE parameters at the gene level.

Let *d* be the proportion of strongly deleterious mutations. We estimated *d* following Mackay *et al.* (2012) as the missing fraction of segregating nonsynonymous sites

(5) $$\hat{d}=1-\frac{P_{N}}{P_{S}}\times \frac{m_{S}}{m_{N}}$$

where *m_S_* and *m_N_* are the total number of synonymous and nonsynonymous sites, respectively.

Let *d_w_* be the fraction of SDM at nonsynonymous sites

(6) $$\hat{d_{w}}=\frac{P_{wd}}{P_{S}}\times \frac{m_{S}}{m_{N}}$$

Lastly, the fraction of effectively neutral mutations *d*_0_ can be estimated as the remaining fraction

(7) $$\hat{d_{0}}=1-d-d_{w}$$

### Properties of the impMKT *α* estimator

We tested the accuracy and performance of the impMKT compared to other MKT approaches at estimating the fraction of substitutions fixed by positive selection (*α*) under different scenarios that were simulated using SLiM 3 (Haller and Messer 2019). The different scenarios considered the combined effects of different genetic features: the level of polymorphism in terms of segregating sites (*θ*), the number of simulated genes, the proportion of adaptive mutations (*p_a_*), the proportion of SDM (*β*), the recombination rate (*ρ*), and the selection strength ($2N_{e}s$) (Table 1). In addition to j>15%, we explored derived allele frequency cutoffs larger than 15% (j>25% and j>35%). We also tested 5% (j>5%) frequency cutoff as in Mackay *et al.* (2012).

**Fig. 2.**
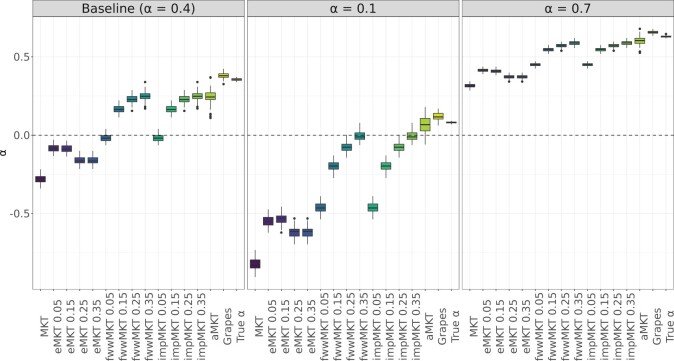
α MKT estimations by the different MKT approaches under different SLiM simulated scenarios, specifically different simulated fractions of adaptive mutations. Equivalent results under other SLiM simulated scenarios are available in Supplementary Fig. 1.

**Fig. 3.**
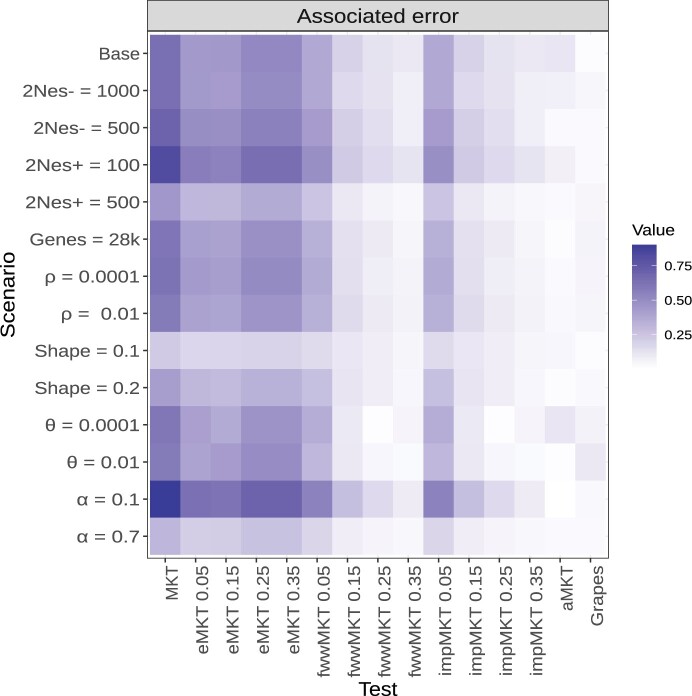
Error biases associated with the *α* estimations for all of the scenarios and MKT approaches.

In all simulations, the original MKT underestimates considerably the *α* values (Figs. 2 and 3 and Supplementary Fig. 1) due to the presence of SDM segregating at low frequencies, excluding simulations where the contribution of SDM is negligible. Overall, the aMKT and Grapes performed better under the presence of SDM and achieved the best results when considering both unbiasedness and efficiency of the estimator (minimum variance) (Fig. 2, Supplementary Fig. 1, and Supplementary Table 2). While heuristic MKT approaches tend to underestimate *α*, Grapes tends to slightly overestimate *α* in most of the scenarios, while aMKT tends to provide slight underestimations (Supplementary Fig. 1 and Supplementary Tables 1 and 2).

As previously shown in Charlesworth and Eyre-Walker (2008), *α* estimates converge to the actual value depending mainly on the shape of the DFE (*β*) and the amount of adaptive evolution (*α*). We considered 3 different values of *β* [0.3 (baseline), 0.2, and 0.1] to test such effect. We observed the same trend for all MKT-derived approaches: the underestimation for the different MKTs is smaller the more leptokurtic DFE is, which in turn implies less SDM. The same effect was found when increasing the rate of adaptive evolution (from α=0.1 to α=0.7, Supplementary Tables 1 and 2 and Fig. 3).

For all the simulated scenarios, the fwwMKT and the impMKT behave similarly to the MKT, mainly depending on the frequency cutoff. As expected, lower cutoffs (i.e. 5%) resulted in minor accuracy improvements in the estimation of *α* compared to the original MKT approach, except when SDM contributed little to the SFS (smaller *β* and larger *α* values). Conversely, larger cutoffs (i.e. 15%, 25%, or 35%) resulted in better estimates of *α*. Specifically, a 35% cutoff is large enough to deal with SDM and to perform estimations similar to the aMKT and Grapes in all simulations. Both the impMKT and the fwwMKT performed very similarly due to the large amount of data considered from the simulations. Contrarily, the eMKT was not able to deal with the presence of SDM and higher frequency cutoffs did not improve the estimations of *α* (Fig. 2, Supplementary Figs. 1–3, and Supplementary Tables 1 and 2; see Discussion).

In scenarios simulating low levels of polymorphism in terms of segregating sites (i.e. reduced number of simulated genes, or reduced mutation rate *θ*), the accuracy and efficiency of the aMKT and Grapes diminishes (Fig. 3, Supplementary Fig. 4, and Supplementary Table 3). Under these circumstances, the aMKT could be applied to approximately 70% of the cases only, and provided worse estimations of *α* than the impMKT. We observe the same trend when measuring the standard deviation of the estimators (Supplementary Fig. 4). impMKT provided better results in comparison to aMKT while showing similar accuracy to Grapes (Supplementary Table 1 and Fig. 3). Similarly, the CIs estimated by Grapes increased by 1 order of magnitude, from range [0.01,0.06] (considering the other scenarios) to 0.16 (for the scenario with 2,000 simulated genes) and 0.19 (for the scenario with θ=0.0001) (Supplementary Fig. 1, 3 and 4 and Fig. 3).

### Estimation of *α* on the presence of recent positive selection

Several studies have showed the contribution of slightly beneficial mutations (SBM) to the SFS at medium/high frequencies over the last years, representing a source of distortion in all MKT approaches. These alleles can segregate in the frequency spectrum and eventually fix in the population depending on the selective strength. Multiple methods have been proposed to overcome this limitation (Galtier 2016; Tataru *et al.* 2017). Nonetheless, many natural patterns remain unanswered, and they can be attributed to the effect of linked selection, since methods that incorporate weak selection assume that sites evolve independently. Uricchio *et al.* (2019) proposed a new MKT approach that incorporates background selection (BGS), estimates the fraction of weak adaptive selection, and discerns the role of linkage in *α* estimations.

We tested such effect following Uricchio *et al.* (2019) simulations to evaluate SBM as well as BGS. We simulated the exact global adaptation rate as it is done in the baseline simulation. As a result, 50% of the *α* signal corresponded to the contribution of weakly advantageous alleles following a point-mass distribution with selection coefficient $2N_{e}s=5$. We chose this selection coefficient after exploring the impact of the population-scaled selection coefficients using the analytical estimations proposed by Uricchio *et al.* (2019). Supplementary Fig. 6 shows that the impact of population-scaled selection coefficients above $2N_{e}s=100$ on the SFS is almost negligible.

In addition to the contribution of SBM to the fixation process, one expects a higher concentration of SBM at high frequencies, since the Hill–Robertson effect prevents them from reaching fixation due to linkage to other SBM or SDM when BGS is acting. Under these assumptions, we modified the impMKT approach to account for such an excess of nonneutral alleles at high frequencies. Similarly to before, we imputed the expected number of SDM at high frequency using the expected $P_{N}/P_{S}$ for low- and high-frequency cutoffs. The expected neutral nonsynonymous/synonymous polymorphism ratio is given by the cutoff *l* and *h*: $P_{N_{\left(l<j<h\right)}}/P_{S_{\left(l<j<h\right)}}$. In this special case, SDM are not removed from the contingency table, but added to the nonsynonymous divergence count. In addition, we executed Grapes using the Gamma-exponential model and considered adaptive mutations using a threshold of $2N_{e}s>5$.

**Fig. 4.**
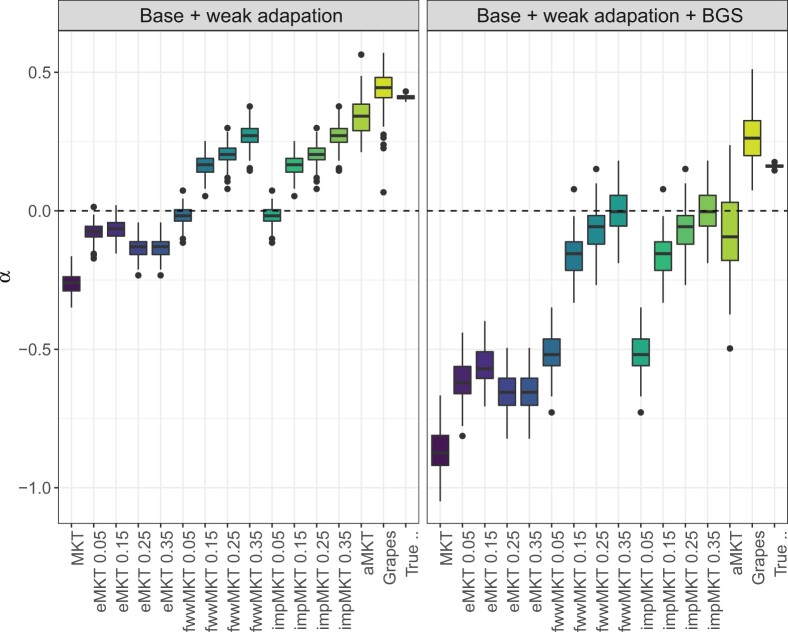
α estimations at simulations accounting for weak adaptation. Any of the proposed methods can correct linkage and weak adaptation at the estimations. Although the method proposed by Uricchio *et al.* (2019) can overcome linkage and weak adaptation, *α* estimations at the gene level remain unexplored and new approaches are required.

In addition to a possible excess at high frequencies, SBM may also segregate all across the spectrum, depending not only on the Hill–Roberston effect but also on the selective strength, the linkage disequilibrium patterns and the fixation times (Supplementary Fig. 6). Assuming that SBM can segregate at any frequency, the impMKT cannot deal with weak adaptation, even imputing nearly fixed variants. Therefore our heuristic approach, extending aMKT results from Uricchio *et al.* (2019), can also be affected by the presence of SBM and BGS. Also Grapes, especially when BGS is acting (Fig. 4 and Supplementary Table 5). All in all, the effect of linkage and the contribution of weak selection at the gene level remain unexplored. Thus, new approaches are needed to pinpoint genes under weak positive selection.

### Testing for the evidence of positive selection in single genes

We estimated *α* at the gene level on *D. melanogaster* (Zambia, ZI; 197 individuals) and human (Africa, AFR; 661 individuals) population data. Table 3 shows the mean values and the number of analyses performed considering different MKT approaches. We removed from the analysis those genes with zero divergence or zero polymorphism, either for synonymous or nonsynonymous sites.

**Table 3.** Gene-by-gene analysis.

Population	Set	MKT		eMKT 0.25		fwwMKT 0.25		impMKT 0.25	
		*N*	*α*	*N*	*α*	*N*	*α*	*N*	*α*
ZI	Analyzable	12,024	−0.721 ± (2.825)	10,340	−0.343 ± (1.582)	7,574	−0.031 ± (1.665)	7,574	−0.031 ± (1.665)
ZI	Negative	1,131	−4.907 ± (7.044)	700	−3.798 ± (3.287)	38	−10.558 ± (10.472)	339	−4.698 ± (4.888)
ZI	Positive	1,493	0.762 ± (0.135)	1,571	0.765 ± (0.134)	929	0.844 ± (0.095)	2,242	0.775 ± (0.121)
AFR	Analyzable	12,786	−1.699 ± (3.206)	6,119	−1.077 ± (2.256)	3,145	−0.686 ± (2.225)	3,145	−0.686 ± (2.225)
AFR	Negative	1,023	−7.433 ± (6.344)	499	−5.259 ± (4.402)	11	−12.695 ± (5.783)	236	−5.471 ± (4.713)
AFR	Positive	76	0.813 ± (0.117)	66	0.794 ± (0.138)	18	0.893 ± (0.093)	203	0.759 ± (0.121)

Total number of analyzable, positively and negatively selected genes by MKT approach.

Due to the amount of raw data, the original MKT was the approach that allowed us to estimate *α* on the largest number of protein-coding genes: 12,024 (87%) genes in the *D. melanogaster* Zambian population. The statistical significance for both positively and negatively selected genes was determined using the Fisher’s exact test; 1,495 and 1,331 were detected under positive and negative selection, respectively. The number of analyzable genes decreased 14% when applying the eMKT correction, from 12,024 to 10,340, but slightly increasing the number of genes under positive selection, from 1,493 to 1,571. We found a decreased of 37% when applying the fwwMKT correction, from 12,024 to 7,574 genes, as well as in the number of genes under positive selection, from 1,495 to 929. More importantly, for both approaches we found a drop in the number of genes under negative selection, from 1,131 to 700 and 38 genes for eMKT and fwwMKT respectively.

**Fig. 5.**
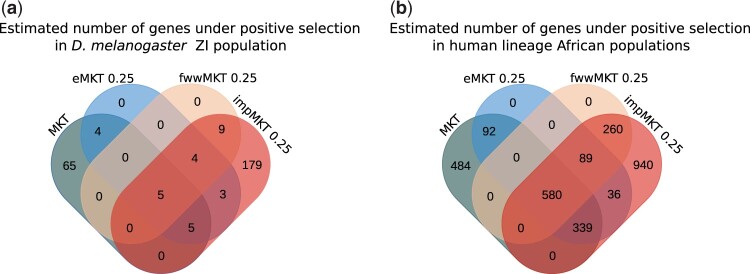
a) Estimated number of genes under positive selection in the *D. melanogaster* Zambian population detected by each MKT approach. b) Estimated number of genes under positive selection in the human lineage African populations detected by each MKT approach.

The impMKT was able to analyze the exact same number of genes as the fwwMKT approach (7,588 genes), since impMKT needs data to compute the $P_{N}/P_{S}$ ratio above the threshold, as the fwwMKT. However, the number of positively selected genes increased from 1,495 in the original MKT approach or 929 in the fwwMKT to 2,244 (Fig. 5a). Therefore, the impMKT increased the detection of positive selection by 50% in the *D. melanogaster* Zambian population compared to the original MKT (1,495 vs. 2,244 genes), by 141% compared to the fwwMKT (929 vs. 2,244) and by 42% regarding eMKT (from 1,571 to 2,242). In addition, the impMKT detected 792% more genes under negative selection than the fwwMKT correction (from 38 in the fwwMKT to 339 genes in the impMKT). We noted a significant drop in the number of genes under negative selection regarding the MKT and eMKT. Nonetheless, since neither MKT nor eMKT is able to deal properly with SDM, as shown in simulations, such trend was not unexpected.

We found similar patterns for the human dataset regarding the MKT and fwwMKT. MKT was the methodology that estimated *α* on the largest number of genes (13,078, 68%), as expected, while fwwMKT and impMKT only analyzed 3,145 genes. Nonetheless, the increase in the number of genes under positive selection detected by the impMKT is especially significant, rising by 159% (from 79 positively selected genes in the MKT to 203 in the impMKT) (Fig. 5b), and the fwwMKT only detected 18 genes under positive selection. Interestingly, contrary to *D. melanogaster* data, eMKT detected less genes than MKT under positive selection. Considering eMKT results from simulations regarding SDM and the associated protein-coding DFE in humans (Booker 2020), we determined that eMKT very sensitive to the underlying DFE.

Overall, in populations with low levels of polymorphism, the impMKT allowed detecting genes under positive selection more efficiently than the other methodologies because it does not remove all the data below a threshold, as the fwwMKT does. By just removing the imputed fraction of SDM, the impMKT can maintain a reasonably good statistical power and, contrary to the fwwMKT, is able to analyze data from datasets with low levels of polymorphism, such as human data. We do not tested aMKT nor Grapes since both methods are not performant or are inaccurate on single-gene sequence data and preferably used in large pools of genes or genome-wide levels.

### Testing for the evidence of positive selection in gene pooling data

Next, we explored the performance of the impMKT, compared to the aMKT, Grapes and the original MKT approach, on pooled gene data. By adding up polymorphism and divergence data from multiple genes, this type of analysis increases the number of polymorphic sites to estimate the SFS, which provides the statistical power necessary to implement both the aMKT and ML approaches. We created gene pools to obtain a reliable measure of the average *α*. Specifically, we first selected 3,500 random protein-coding genes from both the *Drosophila* and the human datasets. Then, we resampled the genes 1,000 times with replacement to create pools of 1, 2, 5, 10, 25, 50, 75, 100, 250, 750, and 1,000 genes on which we computed the SFS and estimated *α* (Table 4).

**Table 4.** *α* estimates by pooled genes.

Bin population	Test	1	2	5	10	25	50	75	100	250	500	750	1,000
ZI	impMKT	−0.015 (−2.332 to 0.915)	0.245 (−1.335 to 0.915)	0.528 (−0.133 to 0.898)	0.596 (0.176 to 0.874)	0.641 (0.403 to 0.828)	0.662 (0.486 to 0.799)	0.67 (0.53 to 0.783)	0.674 (0.557 to 0.774)	0.682 (0.611 to 0.75)	0.684 (0.633 to 0.729)	0.686 (0.647 to 0.722)	0.686 (0.652 to 0.717)
ZI	aMKT	0.888 (0.888 to 0.888)	0.876 (0.865 to 0.894)	0.849 (0.817 to 0.877)	0.661 (0.25 to 0.864)	0.56 (0.17 to 0.821)	0.6 (0.352 to 0.775)	0.628 (0.448 to 0.767)	0.634 (0.482 to 0.762)	0.645 (0.544 to 0.729)	0.65 (0.583 to 0.709)	0.654 (0.595 to 0.703)	0.656 (0.607 to 0.696)
ZI	Grapes	−0.569 (−5.926 to 1.0)	0.4 (−0.843 to 0.992)	0.658 (0.177 to 0.927)	0.704 (0.394 to 0.916)	0.738 (0.551 to 0.876)	0.752 (0.623 to 0.86)	0.758 (0.655 to 0.848)	0.762 (0.672 to 0.839)	0.768 (0.714 to 0.82)	0.769 (0.73 to 0.804)	0.771 (0.74 to 0.799)	0.771 (0.745 to 0.795)
AFR	impMKT	−0.767 (−5.928 to 0.86)	−0.746 (−4.25 to 0.78)	−0.423 (−3.471 to 0.76)	−0.191 (−2.008 to 0.74)	0.023 (−0.862 to 0.6)	0.077 (−0.43 to 0.491)	0.081 (−0.361 to 0.429)	0.093 (−0.269 to 0.39)	0.098 (−0.142 to 0.306)	0.101 (−0.049 to 0.236)	0.101 (−0.015 to 0.213)	0.099 (−0.002 to 0.199)
AFR	aMKT	Nan (nan–nan)	Nan (nan–nan)	Nan (nan–nan)	Nan (nan–nan)	0.487 (0.2 to 0.665)	0.25 (−0.245 to 0.744)	0.086 (−0.555 to 0.573)	0.082 (−0.586 to 0.536)	0.2 (−0.097 to 0.456)	0.189 (0.031 to 0.362)	0.176 (0.051 to 0.327)	0.166 (0.057 to 0.286)
AFR	Grapes	−1.66 (−8.995 to 1.0)	−0.922 (−6.572 to 1.0)	0.119 (−1.155 to 0.954)	0.19 (−0.605 to 0.76)	0.221 (−0.273 to 0.615)	0.237 (−0.121 to 0.537)	0.245 (−0.059 to 0.502)	0.245 (−0.005 to 0.463)	0.257 (0.101 to 0.396)	0.26 (0.152 to 0.353)	0.264 (0.175 to 0.342)	0.265 (0.194 to 0.335)

Each bin number corresponds to the number of pooled genes. Mean estimates and 95 percentiles estimates are shown by MKT approach and bin.

#### *D. melanogaster* ZI population

**Fig. 6.**
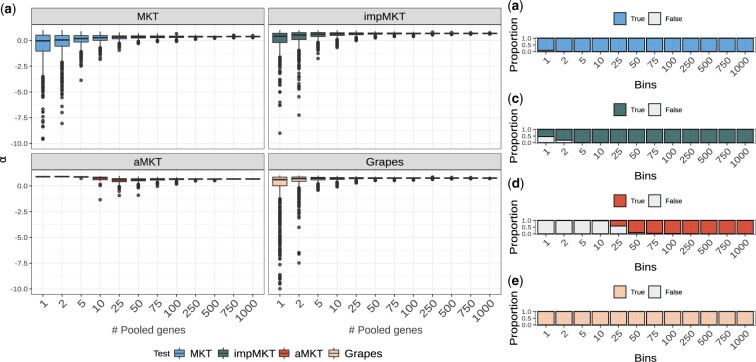
Gene pooled analysis. A total of 3,500 random protein-coding genes were sampled from the ZI dataset. We pooled the genes to obtain SFS of 1, 2, 5, 10, 25, 50, 75, 100, 250, 750, and 1,000 genes by resampling them 1,000 times with replacement. a) *α* estimates with MKT correction. b) Proportion of analysis performed by impMKT. c) Proportion of analysis performed by aMKT. d) Proportion of analysis performed by aMKT. e) Proportion of analysis performed by Grapes.

Resampling analysis results in the *D. melanogaster* ZI population showed that estimated *α* converges to an average value as more and more genes are pooled (Fig. 6a). First, in the case of impMKT, pools of 5 genes or more already allowed estimating *α* in ∼90% of the cases, which corresponds to a mean number of polymorphic sites of *P_N_* = 137 and *P_S_* = 183. A 100% of analyzable cases was achieved in pools of 10 or more genes (Fig. 6b). Second, aMKT required larger pools to analyze the data; pools of 50 genes or more allowed estimating *α* in ∼90% of the cases, which corresponds to a mean number of polymorphic sites of $P_{N}=1,250$ and $P_{S}=1,549$. At least 500 genes were required to estimate *α* in all of the replicates (Fig. 6c).

Third, MKT and Grapes could analyze the vast majority of replicates (except for a few replicates in bins with only 1 or 2 pooled genes). Nonetheless, we noted 1.9-fold (from 1.2 to 2.3) and 8-fold increase (from 1.2 to 9.7) in *α* variance regarding MKT and Grapes compared to impMKT respectively at the first pool, showing the lack of power on small dataset. As the number of genes grow, the mean converging value of *α* was very similar for the impMKT, the aMKT and MKT, and higher for Grapes (Fig. 6a), an expected result considering previous results with simulated data (see previous section). In addition, impMKT showed similar (or higher) *α* values than aMKT and was applicable to the smallest gene pools.

#### Human protein-coding genes

**Fig. 7.**
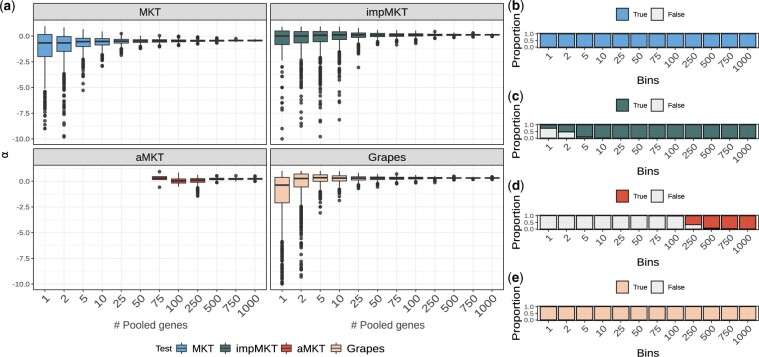
Gene pooled analysis. A total of 3,500 random protein-coding genes were sampled from the human dataset. We pooled the genes to obtain SFS of 1, 2, 5, 10, 25, 50, 75, 100, 250, 750, and 1,000 genes by resampling them 1,000 times with replacement. a) *α* estimates with MKT correction. b) Proportion of analysis performed by impMKT. c) Proportion of analysis performed by aMKT. d) Proportion of analysis performed by aMKT. e) Proportion of analysis performed by Grapes.

Due to the low polymorphism levels in human protein-coding genes compared to *D. melanogaster*, the minimum number of genes pooled to estimate accurate measures of *α* was larger, especially for aMKT (Fig. 7). Specifically, aMKT required pools of 500 genes or more to estimate *α* in ∼90% of the of the replicas, which corresponds to a mean number of polymorphic sites of *P_N_* = 5,658 and *P_S_* = 3,922. More than 1,000 were required to estimate *α* in all of the replicates (Fig. 7c). In the case of Grapes, we found that most of the analyses can be performed; however, they showed a x1.7 increment (from 3.4 to 5.8) increase in the *α* variance regarding impMKT estimations. impMKT could estimate most replicates with 10 or more genes pooled, which corresponds to a mean number of polymorphic sites of *P_N_* = 126 and *P_S_* = 89. All the replicates with 25 or more genes pooled (Fig. 7b) showed similar or higher *α* values than aMKT.

## Discussion

### Effect of SDM on *α* estimation

SDM segregating at low frequencies impact the power of MKT and the estimation of *α* (Templeton 1996; Akashi 1999; Fay *et al.* 2001, 2002; Bustamante *et al.* 2002, 2005; Bierne and Eyre-Walker 2004; Messer and Petrov 2013; Galtier 2016; Rousselle *et al.* 2019). As Bierne and Eyre-Walker (2004) pointed out, unless the methodology considers the presence of SDM, estimations using *D. melanogaster* data are likely underestimating *α*. We verify such statements by thoroughly exploring the MKT-derived approaches using both *in silico* and empirical data, assessing the benefits and drawbacks of each methodology, considering the nature of the data and the study design. Simulations with SLiM 3 have been carried out to benchmark the performance of the 4 MKT methodologies and the impMKT under different evolutionary scenarios. Predefined *α* values were used to assess the closest estimation. aMKT and Grapes are the best methods with respect to unbiasedness and efficiency of estimated values of *α*. However, their performance decreases in scenarios with a small number of polymorphic variants (shorter genomic regions or lower mutation rate) or could not even be applied due to low variant counts. Our results are consistent with previous explorations of MKT-derived approaches (Charlesworth and Eyre-Walker 2008; Messer and Petrov 2013). Hence, we found similar results exploring aMKT and Grapes in Drosophila and human genome sequence data and showed similar accuracy in simulations. Overall, both approaches allow efficient removal of SDM in all frequencies and not only below a threshold as in fwwMKT or impMKT methods.

Strikingly, both procedures lack power when applied to individual genes or small pooled datasets. Despite the high polymorphic and divergence levels in *D. melanogaster*, it is not enough for the aMKT to fit the exponential curve and calculate *α* for single genes, and the number of analyzable genes is dramatically reduced (see Table 3). We showed that pooled sets of genes allow overcoming data limitations to estimate an overall *α* value (Boyko *et al.* 2008; Eyre-Walker and Keightley 2009). Thus we explored the minimum number of pooled genes to perform aMKT regarding *D. melanogaster* and human populations. For aMKT we found that a minimum of 500 genes is required to perform 1,000 replicas when bootstrapping a set of 3,500 random genes (Fig. 6). Such a number increased to more than 1,000 when using the human dataset (Fig. 7). We found that Grapes can perform the estimation most of the time (only a few negligible analyses were not performed, see Figs. 6 and 7), considering gene-by-gene analysis or pooled analysis. Nonetheless, we found extremely high variance in *α* estimates and we noted that the associated CI to *α* estimation for each bootstrapped datasets is only acceptable once the analysis accounts for a minimum number of 50 in Drosophila and humans (see Supplementary Fig. 8). The same trend is observed in those simulated scenarios producing less polymorphism regarding the percentage of aMKT analysis and Grapes CIs (Supplementary Figs. 1 and 3). Given the high levels of polymorphism in *D. melanogaster* compared to humans, the similar results for both populations can be considered as general ones.

Such findings show the limitation of aMKT and Grapes (and other ML methods) (Eyre-Walker and Keightley 2009; Racimo and Schraiber 2014; Tataru *et al.* 2017) when performing MKT at the gene-by-gene level or using small pooled datasets. Among non-ML approaches, fwwMKT and impMKT produced quite similar results. However, only when using higher frequency cutoffs than the commonly used 15% they showed results close to those by aMKT and Grapes (see Table 2 and Supplementary Table 2). Such cutoffs can be astringent considering empirical data, especially in the case of fwwMKT. Instead of removing all polymorphism at low frequencies at both synonymous and nonsynonymous sites, as fwwMKT does, the new impMKT separates *P_N_* into the number of effectively neutral variants and the number of SDM, and only removes the latter. In this way, impMKT allows increasing the frequency cutoff without compromising the amount of data that much. As a result, impMKT is the most powerful method to detect selection at the gene-by-gene level, substantially increasing the number of statistically significant genes under positive selection compared to other methodologies (see Fig. 5 and Table 3). In the case of pooled analyses, impMKT reduced dramatically the minimum number of genes required to perform the analysis in both Drosophila and human datasets (5 and 10, respectively).

Even though strongly deleterious (*d*), slightly deleterious (*d_w_*) and effectively neutral (*d*_0_) mutations are commonly defined given DFE ranges $-10>N_{e}s,-10<N_{e}s<-1$, and $-1<N_{e}s<1$, respectively, we observed mutations segregating in the range $-10<N_{e}s$. Hence, if *d* is the proportion of mutations not segregating because of strong purifying selection, as stated above, we estimated *d_w_* including any segregating mutation below the threshold $N_{e}s<-1$. Supplementary Table 4 and Supplementary Fig. 5 show impMKT unbiased estimations of *d*, *d_w_* and *d*_0_ using 5% and 35% cutoffs. Similarly to *α* estimation, the estimator requires larger cutoff than 5–15% (Charlesworth and Eyre-Walker 2008; Mackay *et al.* 2012) to properly impute SDM and estimate *d_w_* and *d*_0_ accurately. Hence, the new impMKT provides easier and faster estimations of *d*, *d_w_*, and *d*_0_ than ML approaches, representing the actual mutation proportions subject to different selection regimes and quantitative measures of the DFE along the genome or at the gene level.

### The effect of pooling data

We showed that most MKT approaches could provide an accurate estimate of the average *α* if data from a large number of genes are collected (Hahn 2018). Therefore, the process of pooling genes to create single evolutionary entities is a proper strategy to overcome the problem of lacking enough polymorphism data to conduct an MKT. In the majority of the performed analyses, this process does not seem to affect the results. However, some caveats must be taken into account when interpreting results obtained by this procedure.

First, pooled genes do not necessarily share the same recombination context, GC content, or gene density rate, which also affect the adaptive potential of genes. Although pooling genes by 1 or more features at a time have been widely used to disentangle the potential drivers of adaptation (Castellano *et al.* 2016; Moutinho *et al.* 2019; Uricchio *et al.* 2019; Rousselle *et al.* 2019; Soni *et al.* 2021), such approaches can report a spurious association between adaptation signals and other features if they are strongly correlated (Huang 2021). Huang (2021) developed the so-called MK regression to overcome biases of pooling analyses applied to 1 genomic feature at a time, by jointly evaluating the effects of correlated genomic features on *α* estimation. Nonetheless, MK regression is designed to measure the adaptation rate at the genomic level, and consequently not the preferred approach to pinpoint individual genes neither (Huang 2021). Interestingly, we have noticed that MK regression followed the strategy proposed by Fay *et al.* (2001) to deal with SDM. We propose to apply our impMKT approach instead, to preserve data and extending the implementation at the gene-by-gene level.

Second, by pooling hundreds of genes, it is more difficult to detect a signal of positive selection if it is due to a few genes of the pool. In other words, all the evolutionary forces acting differently on different genes contribute to the dilution of potential biological signals.

Third, although this data pooling increases the power of detecting selection, it could lead to the Simpson’s paradox (Simpson 1951) if a significant trend in the 2 × 2 contingency tables disappears or reverses when the data is combined into a single table (Stoletzki and Eyre-Walker 2011; Hahn 2018). Regarding MKT data, this can happen when large differences in the number of nonsynonymous fixations (*D_N_*) between genes lead to incorrect inferences about selection operating in different regions (Stoletzki and Eyre-Walker 2011).

### Folded SFS vs. unfolded SFS

Because the unfolded SFS (uSFS) provides more evolutionary information, all the analyses shown here take advantage of it. Nonetheless, uSFS needs that ancestral alleles are precisely estimated. The inference of ancestral states requires genetic data for at least 1 outgroup species and the application of ML methods. Because we used a parsimony approach with a single outgroup to estimate the uSFS, there may be polarization errors affecting our empirical analyses. The misattribution of ancestral alleles can also affect *α* and the MKT estimations (Hernandez *et al.* 2007). On the one hand, an excess of high-frequency alleles can be attributed to hitchhiking of linked selected alleles or weak adaptation, which can affect ML methods that infer the DFE by over-estimating the role of positive selection. On the other hand, an excess of high-frequency alleles will specially affect the asymptotic fit in the aMKT, resulting in an under-estimation of *α*. To date, the method proposed by Keightley and Jackson (2018) is the most sophisticated approximation to estimate the uSFS while minimizing mispolarization errors. The method uses the genetic data of 2 or more outgroup species, considers their phylogenetic tree topology, and considers multiple nucleotide substitution models.

Nonetheless, because having genetic data of outgroup species is not always possible, we explored how the impMKT performs on folded SFS (fSFS) data instead of uSFS. The fSFS analyses causes a slight, affordable, decrease in the mean estimates of *α* (see Supplementary Fig. 9), which is more pronounced as the frequency cutoff increases. It should be noticed that by applying the frequency cutoff on the fSFS, both the low-frequency and high-frequency derived alleles are removed from the analyses, which reduces the data for the estimation of *α* (and thus the statistical power). The same trend occurs when using the fSFS in gene-by-gene analyses for both *Drosophila* and humans, decreasing the number of positively selected genes by 25% and 44%, respectively (see Supplementary Table 6).

Nevertheless, using the fSFS and focusing only on the central part of the frequency spectrum can be especially interesting in 2 cases. First, it is a better choice when mispolarization errors are abundant, a situation which would add an additional bias to SDM. The cutoff will potentially eliminate fictitious derived alleles (due to mispolarization) at a high frequency that would deviate the ratio $P_{N(j>15\%)}/P_{S(j>15\%)}$ used in the imputation. Second, the cutoff will eliminate the accumulation of SDM at high frequencies due to interference between positively selected and slightly deleterious alleles.

## Supplementary Material

jkac206_Supplementary_Data

## Data Availability

Human and *D. melanogaster* processed data and the new impMKT software implementation are available at imkt.uab.cat (Murga-Moreno *et al.* 2019). The supporting figures as well as notebooks and code used to perform the analyses can be found at https://github.com/jmurga/mkt_comparison. Supplemental material is available at *G3* online.
