# Supplementary material for: impMKT: the imputed McDonald and Kreitman test, a straightforward correction that significantly increases the evidence of positive selection of the McDonald and Kreitman test at the gene level
Source: G3 (Bethesda). 2022 Aug 17;12(10):jkac206. doi: 10.1093/g3journal/jkac206 (PMC9526038; doi:10.1093/g3journal/jkac206)
Supplement: jkac206_Supplementary_Data [file jkac206_supplementary_data.pdf]

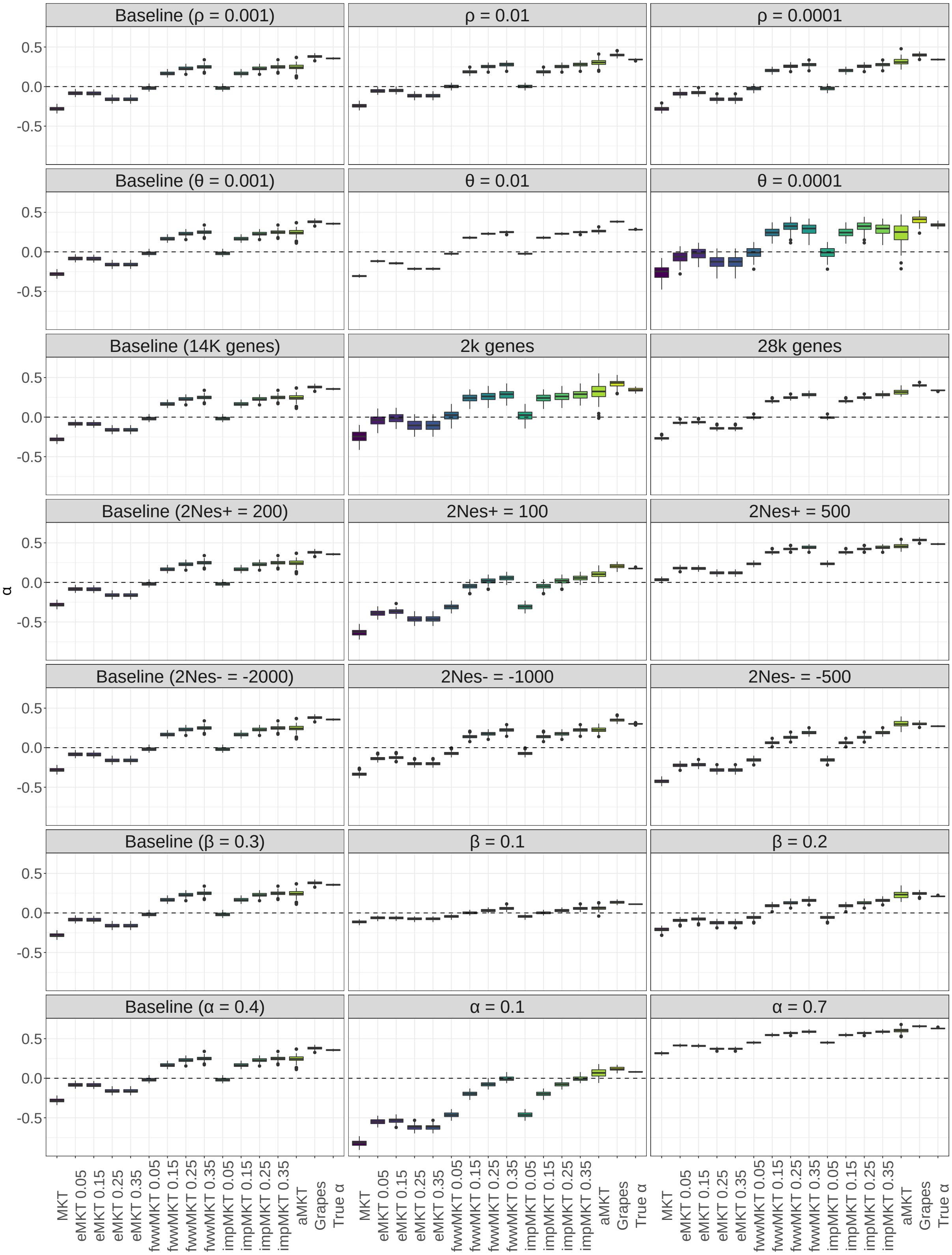

Figure 1 α MKT estimations on different SLiM simulated scenarios. ρ and θ are the population-scaled recombination and mutation rates ($\rho = 4N_e r$, $\theta = 4N_e\mu$, respectively). $2N_e s$ is the scaled-population selection coefficient for beneficial and deleterious alleles. β is the shape parameter of the Gamma DFE for selected alleles.

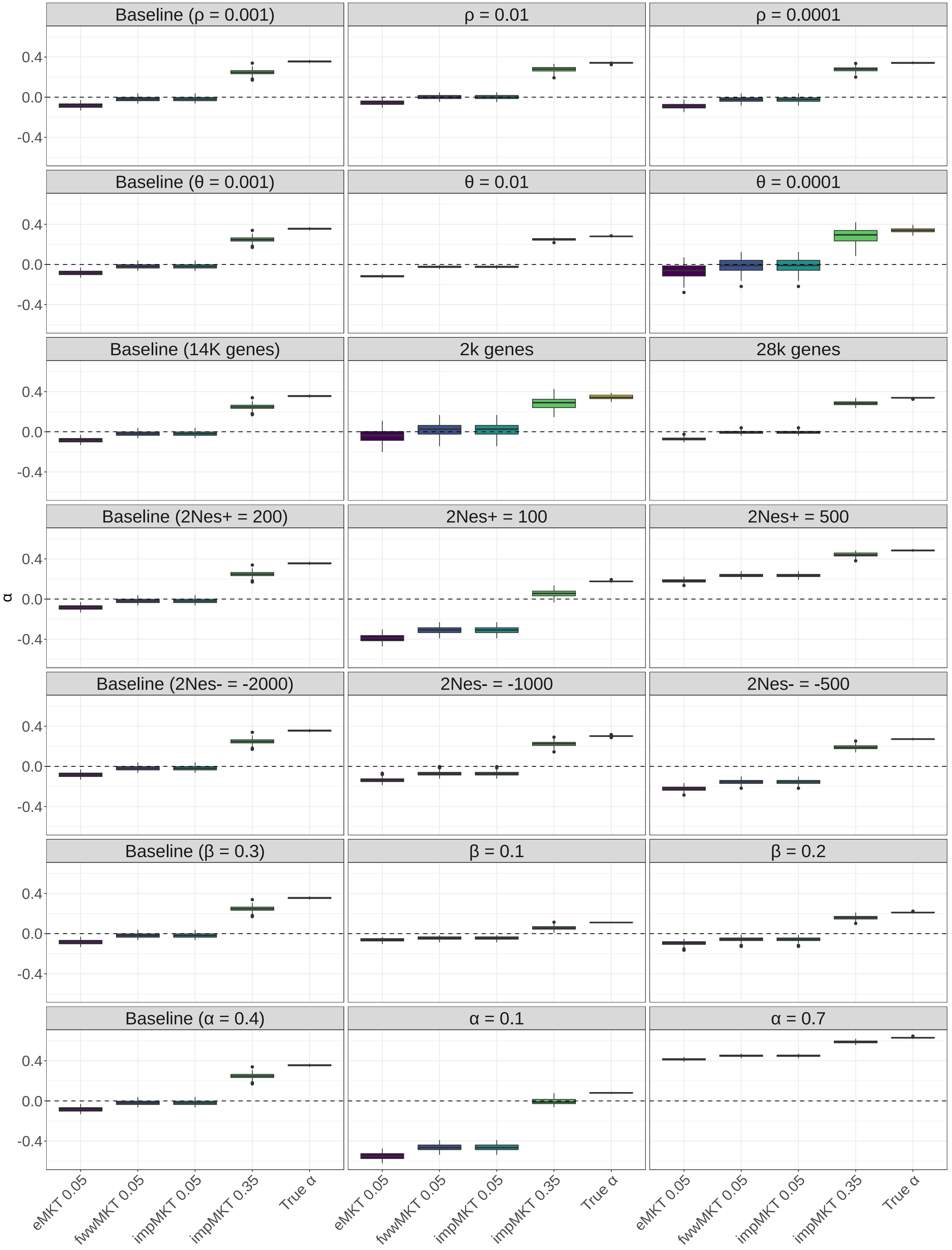

Figure 2 impMKT α estimations using different cutoff

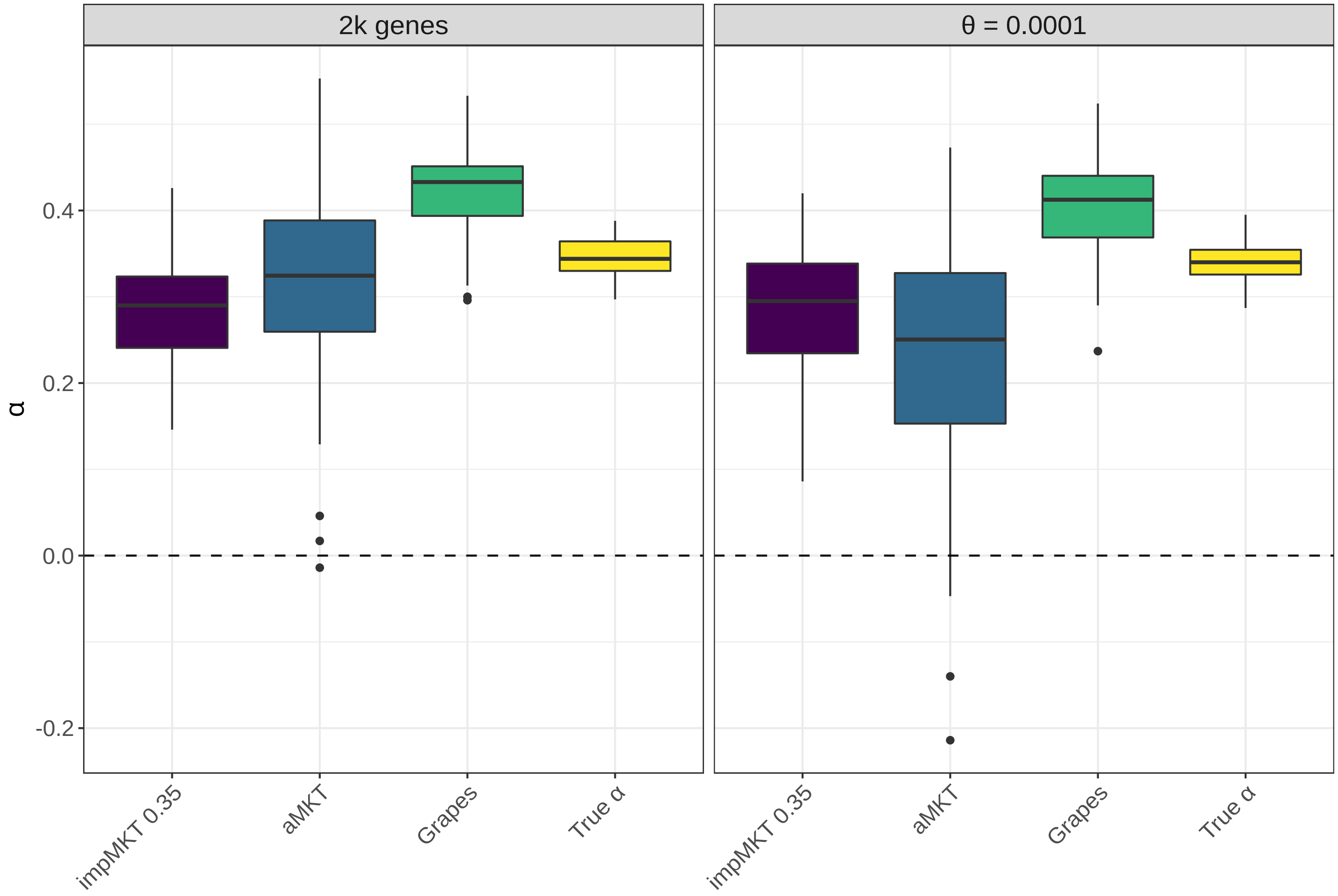

Figure 3 Effect on α estimation reducing of the amount of segregating sites (i.e., reducing the mutation rate θ to 0.0001, and reducing the total number of simulated genes to 2000) for different MKT approaches.

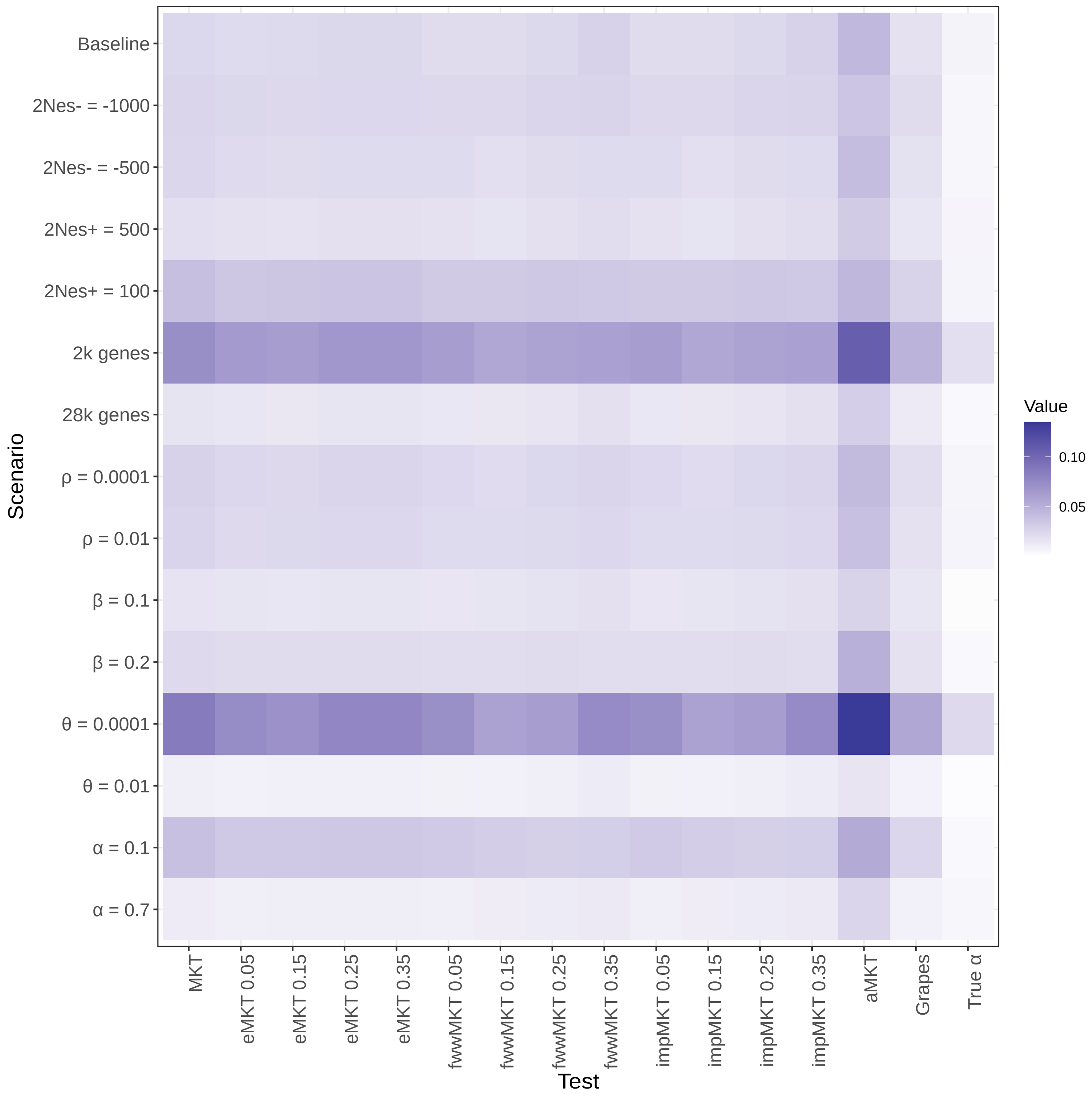

Figure 4 Standard deviation heatmap for each scenario and MKT approach

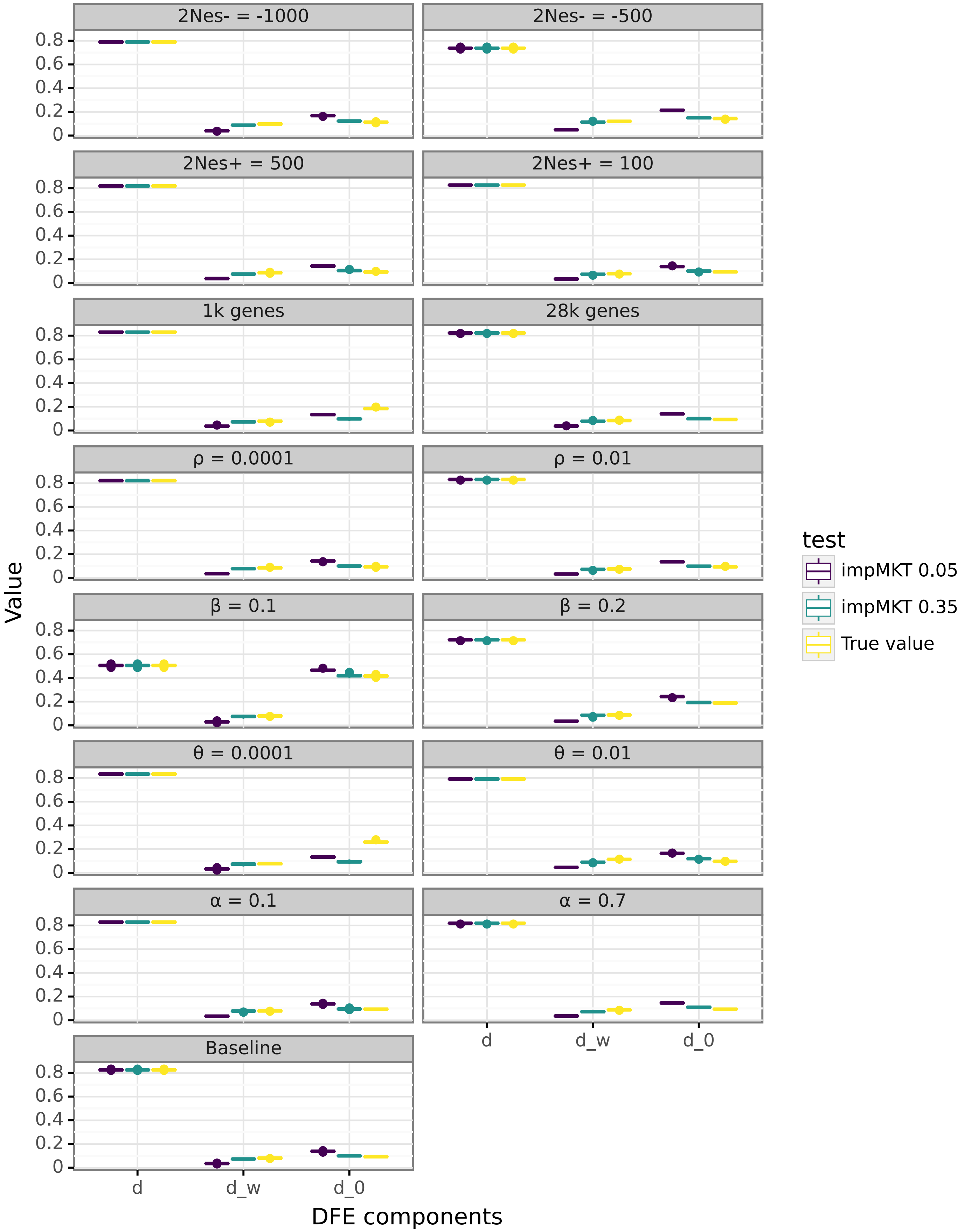

Figure 5 d, d_w and d_0 impMKT estimations on different SLiM simulated scenarios. ρ and θ are the population-scaled recombination and mutation rates ($\rho = 4N_e r$, $\theta = 4N_e \mu$). $2N_e s$ is the scaled-population selection coefficient for beneficial and deleterious alleles. β is the shape parameter of the gamma DFE.

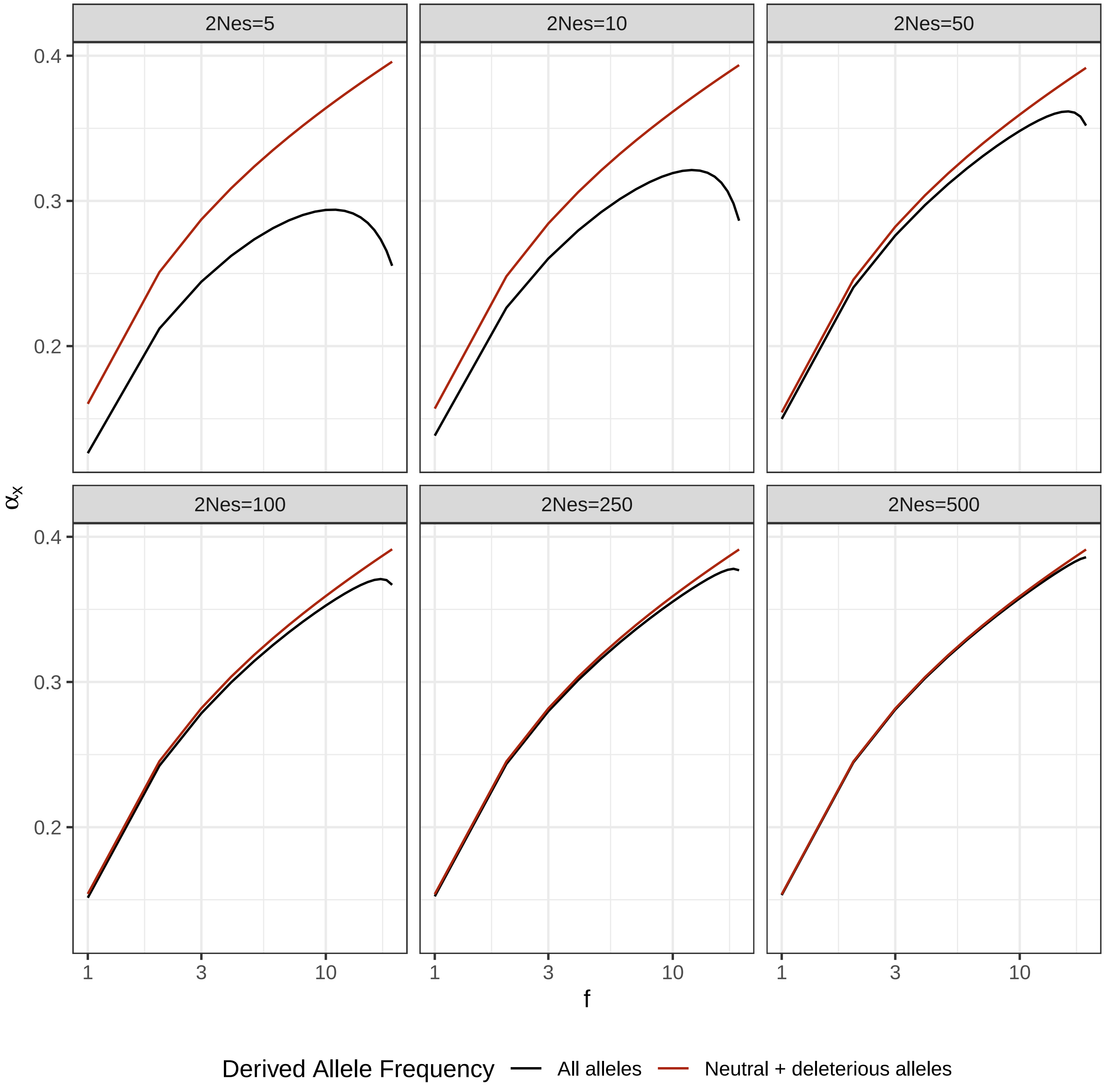

Figure 6 α_x estimation obtained by incorporating a weak adaptation component. Results show that 50% of α corresponded to the contribution of weakly advantageous alleles while assuming different selection coefficients.

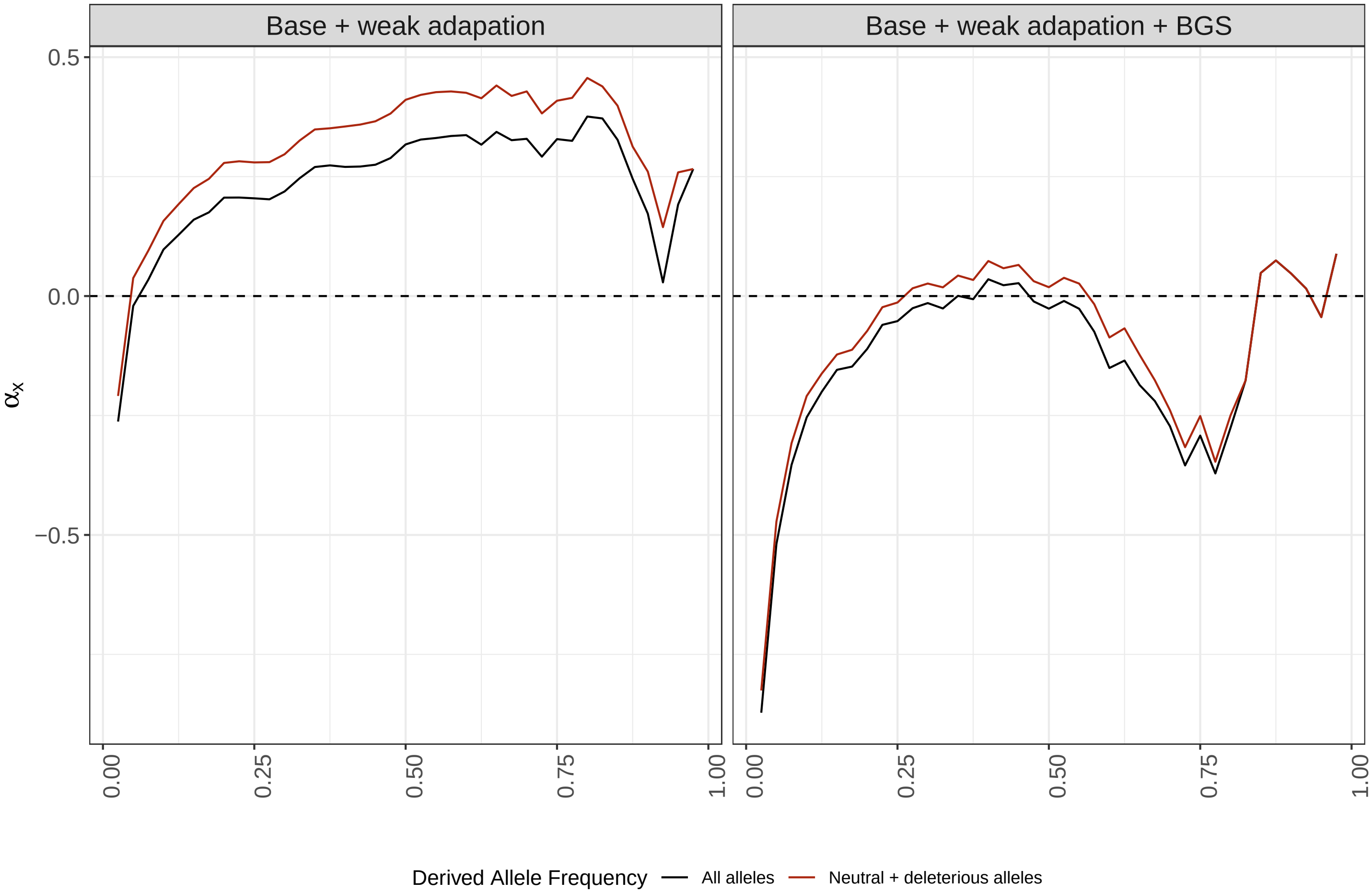

Figure 7 Effect on estimated α_x of adding BGS and weak adaptation to the baseline simulation for each frequency category of the SFS. Weakly beneficial variants tend to segregate along with the SFS. Therefore impMKT cannot deal with weak adaptation even when a cutoff is set to remove high frequency alleles.

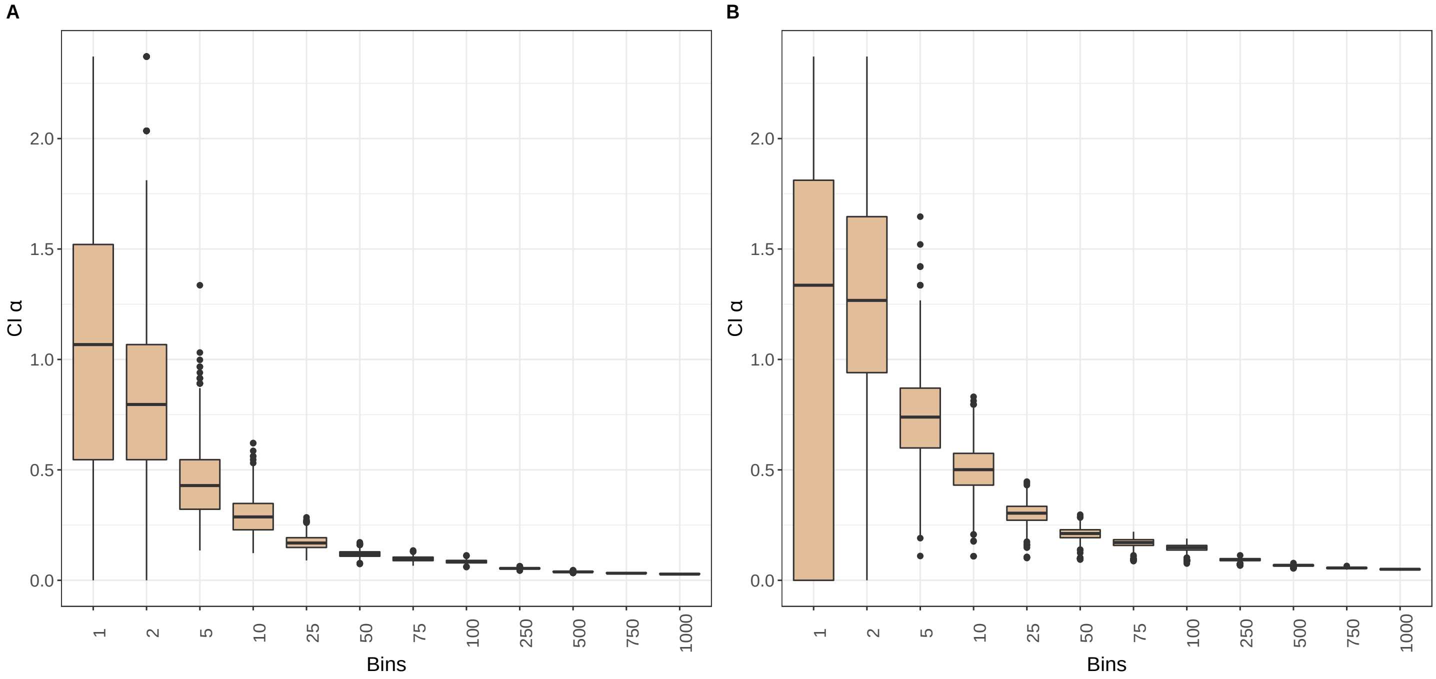

Figure 8 Associated CI to α estimation of 1000 replicates when bootstrapping a set of 3500 random genes. A. *D. melanogaster* gene pooled dataset. B Human gene pooled dataset.

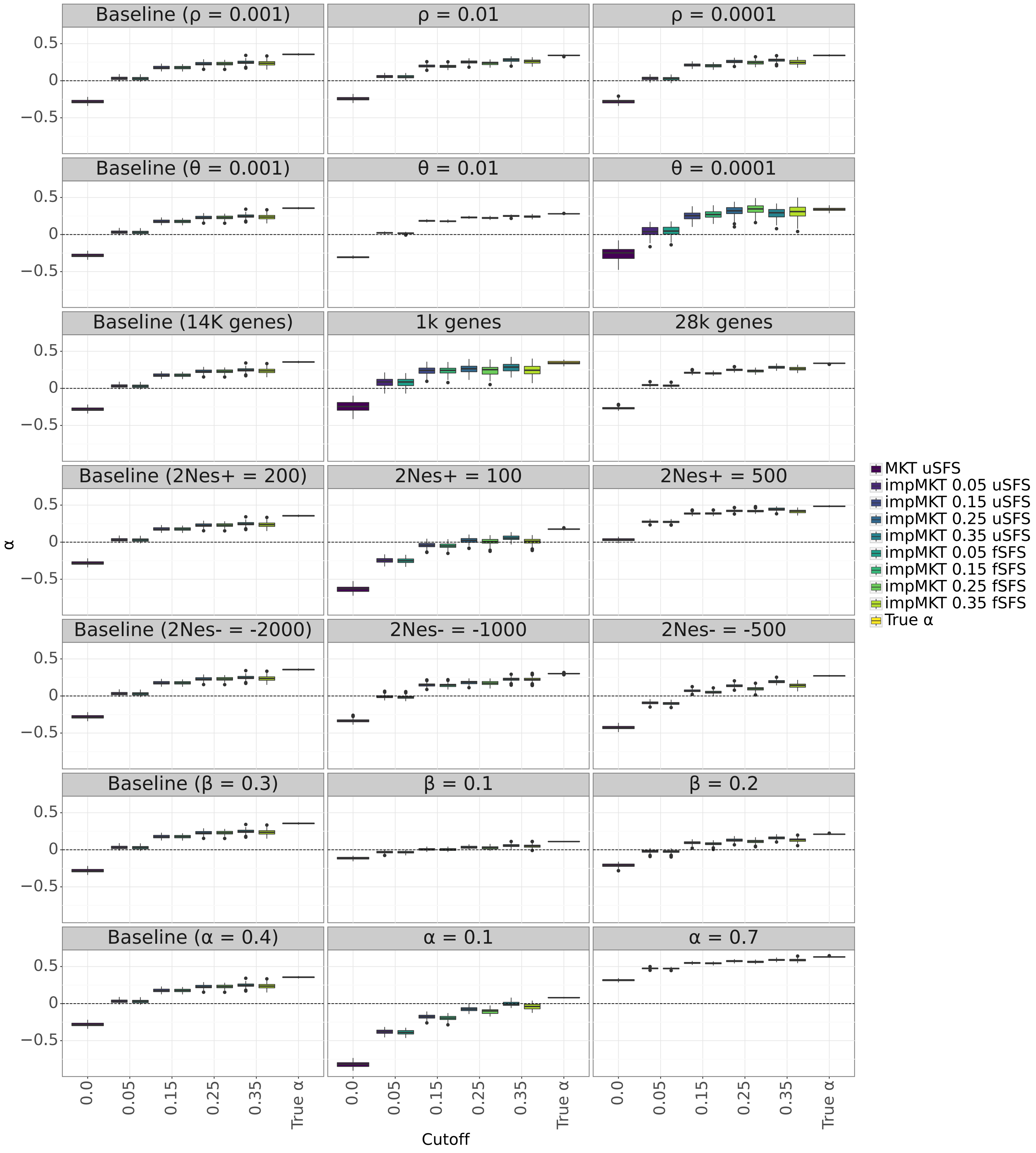

Figure 9 Replicates of the analysis performed in section *Properties of the impMKT α estimator*. We used the fSFS and the uSFS to test the impMKT.

Table 1 Mean α values for each scenario and MKT approach. Mean values were calculated using bootstrap distributions.

Simulations	MKT	eMKT 0.05	eMKT 0.15	eMKT 0.25	eMKT 0.35	fwwMKT 0.05	fwwMKT 0.15	fwwMKT 0.25	fwwMKT 0.35	impMKT 0.05	impMKT 0.15	impMKT 0.25	impMKT 0.35	aMKT	Grapes	True
Baseline	-0.284	-0.086	-0.088	-0.162	-0.162	-0.019	0.167	0.229	0.251	-0.019	0.167	0.229	0.251	0.233	0.425	0.356
$2N_es- = -1000$	-0.33	-0.134	-0.12	-0.199	-0.199	-0.07	0.144	0.181	0.225	-0.07	0.144	0.181	0.225	0.243	0.406	0.301
$2N_es- = -500$	-0.423	-0.22	-0.212	-0.28	-0.28	-0.152	0.06	0.128	0.188	-0.152	0.06	0.128	0.188	0.269	0.361	0.271
$2N_es+ = 100$	-0.63	-0.385	-0.364	-0.457	-0.457	-0.306	-0.044	0.023	0.06	-0.306	-0.044	0.023	0.06	0.071	0.283	0.177
$2N_es+ = 500$	0.033	0.18	0.175	0.12	0.12	0.235	0.38	0.42	0.439	0.235	0.38	0.42	0.439	0.438	0.571	0.485
Genes 2000	-0.241	-0.037	-0.01	-0.102	-0.102	0.024	0.246	0.263	0.288	0.024	0.246	0.263	0.288	0.293	0.49	0.349
Genes 28000	-0.269	-0.072	-0.064	-0.142	-0.142	-0.006	0.203	0.248	0.285	-0.006	0.203	0.248	0.285	0.316	0.46	0.338
$\rho = 0.0001$	-0.283	-0.091	-0.08	-0.162	-0.162	-0.025	0.202	0.255	0.278	-0.025	0.202	0.255	0.278	0.295	0.461	0.34
$\rho = 0.01$	-0.239	-0.054	-0.044	-0.111	-0.111	0.003	0.192	0.259	0.283	0.003	0.192	0.259	0.283	0.285	0.454	0.344
$\beta = 0.1$	-0.114	-0.064	-0.065	-0.075	-0.075	-0.047	-0.001	0.027	0.053	-0.047	-0.001	0.027	0.053	0.055	0.123	0.111
$\beta = 0.2$	-0.212	-0.098	-0.08	-0.125	-0.125	-0.061	0.087	0.124	0.157	-0.061	0.087	0.124	0.157	0.224	0.298	0.21
$\theta = 0.0001$	-0.254	-0.061	-0.02	-0.12	-0.12	-0.003	0.244	0.327	0.293	-0.003	0.244	0.327	0.293	0.189	0.479	0.339
$\theta = 0.01$	-0.306	-0.116	-0.144	-0.214	-0.214	-0.022	0.181	0.23	0.25	-0.022	0.181	0.23	0.25	0.256	0.427	0.28
$\alpha = 0.1$	-0.821	-0.547	-0.536	-0.619	-0.619	-0.46	-0.198	-0.079	-0.006	-0.46	-0.198	-0.079	-0.006	0.06	0.199	0.08
$\alpha = 0.7$	0.317	0.415	0.409	0.373	0.373	0.452	0.548	0.571	0.589	0.452	0.548	0.571	0.589	0.596	0.678	0.63

Table 2 Mean error bias for each scenario and MKT approach. Error bias were measured through the difference of mean values of α for each method and the true value of α.

Simulations	MKT	eMKT 0.05	eMKT 0.15	eMKT 0.25	eMKT 0.35	fwwMKT 0.05	fwwMKT 0.15	fwwMKT 0.25	fwwMKT 0.35	impMKT 0.05	impMKT 0.15	impMKT 0.25	impMKT 0.35	aMKT	Grapes	
Baseline	0.638	0.441	0.443	0.517	0.517	0.374	0.189	0.127	0.106	0.374	0.189	0.127	0.106	0.111	0.025	
$2N_es_- = -1000$	0.634	0.438	0.425	0.502	0.502	0.373	0.161	0.125	0.079	0.373	0.161	0.125	0.079	0.074	0.049	
$2N_es_- = -500$	0.698	0.493	0.485	0.553	0.553	0.425	0.21	0.141	0.081	0.425	0.21	0.141	0.081	0.03	0.028	
$2N_es_+ = 100$	0.812	0.565	0.546	0.639	0.639	0.487	0.226	0.159	0.122	0.487	0.226	0.159	0.122	0.073	0.029	
$2N_es_+ = 500$	0.451	0.304	0.308	0.363	0.363	0.249	0.102	0.062	0.042	0.249	0.102	0.062	0.042	0.027	0.053	
Genes = 2k	0	0.589	0.386	0.36	0.451	0.451	0.325	0.108	0.087	0.063	0.325	0.108	0.087	0.063	0.024	0.079
Genes = 28k	0	0.606	0.41	0.402	0.48	0.48	0.344	0.136	0.091	0.055	0.344	0.136	0.091	0.055	0.021	0.063
$\rho = 0.0001$	0.624	0.432	0.42	0.503	0.503	0.365	0.138	0.085	0.063	0.365	0.138	0.085	0.063	0.027	0.058	
$\rho = 0.01$	0.585	0.4	0.391	0.458	0.458	0.343	0.156	0.09	0.066	0.343	0.156	0.09	0.066	0.037	0.055	
$\beta = 0.1$	0.224	0.173	0.175	0.185	0.185	0.157	0.11	0.082	0.055	0.157	0.11	0.082	0.055	0.051	0.023	
$\beta = 0.2$	0.42	0.306	0.288	0.334	0.334	0.268	0.12	0.083	0.051	0.268	0.12	0.083	0.051	0.022	0.037	
$\theta = 0.0001$	0.6	0.408	0.364	0.467	0.467	0.35	0.098	0.018	0.057	0.35	0.098	0.018	0.057	0.113	0.066	
$\theta = 0.01$	0.586	0.397	0.425	0.494	0.494	0.304	0.101	0.051	0.031	0.304	0.101	0.051	0.031	0.017	0.102	
$\alpha = 0.1$	0.901	0.629	0.616	0.699	0.699	0.543	0.278	0.158	0.087	0.543	0.278	0.158	0.087	0.014	0.038	
$\alpha = 0.7$	0.313	0.216	0.221	0.257	0.257	0.179	0.083	0.059	0.041	0.179	0.083	0.059	0.041	0.027	0.028	

Table 3 Standard deviation value for each scenario and MKT approach.

Simulations	MKT	eMKT 0.05	eMKT 0.15	eMKT 0.25	eMKT 0.35	fwwMKT 0.05	fwwMKT 0.15	fwwMKT 0.25	fwwMKT 0.35	impMKT 0.05	impMKT 0.15	impMKT 0.25	impMKT 0.35	aMKT	Grapes	True
Baseline	0.0253	0.0224	0.0231	0.0245	0.0245	0.0217	0.0221	0.0239	0.0282	0.0217	0.0221	0.0239	0.0282	0.0448	0.0184	0.0075
$2N_es- = 1000$	0.0264	0.0247	0.0243	0.0252	0.0252	0.0244	0.0243	0.0263	0.0271	0.0244	0.0243	0.0263	0.0271	0.0364	0.0219	0.0058
$2N_es- = 500$	0.0254	0.0231	0.0217	0.0227	0.0227	0.0228	0.0197	0.0220	0.0228	0.0228	0.0197	0.0220	0.0228	0.0417	0.0189	0.0053
$2N_es+ = 100$	0.0201	0.0188	0.0181	0.0190	0.0190	0.0187	0.0175	0.0191	0.0217	0.0187	0.0175	0.0191	0.0217	0.0327	0.0155	0.0067
$2N_es+ = 500$	0.0407	0.0350	0.0362	0.0369	0.0369	0.0336	0.0337	0.0347	0.0338	0.0336	0.0337	0.0347	0.0338	0.0452	0.0273	0.0068
Genes 2000	0.0727	0.0647	0.0630	0.0667	0.0667	0.0629	0.0559	0.0589	0.0608	0.0629	0.0559	0.0589	0.0608	0.1071	0.0480	0.0197
Genes 28000	0.0170	0.0154	0.0152	0.0161	0.0161	0.0150	0.0151	0.0166	0.0193	0.0150	0.0151	0.0166	0.0193	0.0307	0.0130	0.0046
$\rho = 0.0001$	0.0281	0.0249	0.0245	0.0261	0.0261	0.0242	0.0224	0.0252	0.0263	0.0242	0.0224	0.0252	0.0263	0.0430	0.0203	0.0065
$\rho = 0.01$	0.0268	0.0235	0.0240	0.0251	0.0251	0.0227	0.0224	0.0233	0.0251	0.0227	0.0224	0.0233	0.0251	0.0395	0.0185	0.0070
$\beta = 0.1$	0.0171	0.0160	0.0155	0.0164	0.0164	0.0159	0.0163	0.0183	0.0194	0.0159	0.0163	0.0183	0.0194	0.0273	0.0156	0.0021
$\beta = 0.2$	0.0237	0.0218	0.0217	0.0219	0.0219	0.0215	0.0214	0.0217	0.0216	0.0215	0.0214	0.0217	0.0216	0.0506	0.0187	0.0046
$\theta = 0.0001$	0.0858	0.0740	0.0702	0.0776	0.0776	0.0716	0.0599	0.0629	0.0749	0.0716	0.0599	0.0629	0.0749	0.1342	0.0559	0.0236
$\theta = 0.01$	0.0103	0.0094	0.0097	0.0101	0.0101	0.0090	0.0094	0.0104	0.0125	0.0090	0.0094	0.0104	0.0125	0.0165	0.0084	0.0019
$\alpha = 0.1$	0.0395	0.0343	0.0340	0.0343	0.0343	0.0333	0.0316	0.0302	0.0304	0.0333	0.0316	0.0302	0.0304	0.0541	0.0255	0.0045
$\alpha = 0.07$	0.0122	0.0106	0.0112	0.0114	0.0114	0.0103	0.0115	0.0127	0.0137	0.0103	0.0115	0.0127	0.0137	0.0266	0.0092	0.0054

Table 4 Mean error bias for each scenario and MKT approach. Error bias were measured through the difference of mean values of d, d_w and d_0 for each method and the true value retrieved from `SLiM`.

Analysis	ImpMKT 0.05			ImpMKT 0.05		
	d	d_w	d_0	d	d_w	d_0
Baseline	4.43E-10	4.53E-02	4.51E-02	3.52E-10	8.08E-03	7.89E-03
$2N_es- = 1000$	5.40E-10	5.67E-02	5.64E-02	2.09E-10	1.02E-02	9.93E-03
$2N_es- = 500$	4.98E-11	7.00E-02	6.97E-02	1.23E-10	7.50E-03	7.14E-03
$2N_es+ = 100$	7.12E-10	4.49E-02	4.48E-02	2.97E-10	6.04E-03	5.91E-03
$2N_es+ = 500$	7.04E-10	4.95E-02	4.93E-02	5.61E-10	1.15E-02	1.13E-02
2000 genes	4.04E-09	4.15E-02	5.09E-02	8.43E-10	5.11E-03	8.73E-02
28000 genes	4.47E-10	4.76E-02	4.75E-02	4.49E-10	6.86E-03	6.80E-03
$\rho = 0.0001$	8.83E-10	4.92E-02	4.89E-02	1.88E-10	6.91E-03	6.53E-03
$\rho = 0.01$	2.83E-10	4.35E-02	4.32E-02	1.33E-10	4.95E-03	4.63E-03
$\beta = 0.1$	3.67E-10	4.99E-02	4.82E-02	3.03E-10	4.53E-03	2.83E-03
$\beta = 0.2$	8.56E-11	5.42E-02	5.33E-02	3.41E-10	4.20E-03	3.23E-03
$\theta = 0.0001$	4.93E-09	4.37E-02	1.26E-01	1.12E-08	4.31E-03	1.65E-01
$\theta = 0.01$	3.51E-11	6.77E-02	6.77E-02	9.04E-11	2.41E-02	2.40E-02
$\alpha = 0.1$	3.37E-10	4.52E-02	4.46E-02	6.15E-10	2.38E-03	1.86E-03
$\alpha = 0.7$	3.14E-10	5.25E-02	5.33E-02	7.40E-12	1.53E-02	1.61E-02

Table 5 Mean α values from simulations accounting for weak adaptation and BGS.

Simulations	MKT	eMKT 0.05	eMKT 0.15	eMKT 0.25	eMKT 0.35	fwwMKT 0.05	fwwMKT 0.15	fwwMKT 0.25	fwwMKT 0.35	impMKT 0.05	impMKT 0.15	impMKT 0.25	impMKT 0.35	aMKT	Grapes	True
Baseline	-0.282	-0.085	-0.088	-0.161	-0.161	-0.019	0.166	0.228	0.249	-0.019	0.166	0.228	0.249	0.245	0.381	0.355
Baseline + weak adaptation	-0.261	-0.077	-0.068	-0.134	-0.134	-0.019	0.164	0.203	0.27	-0.019	0.164	0.203	0.27	0.345	0.434	0.41
Baseline + weak adaptation + BGS	-0.866	-0.615	-0.56	-0.654	-0.654	-0.515	-0.159	-0.063	-0.003	-0.515	-0.159	-0.063	-0.003	-0.096	0.267	0.161

Table 6 Number of detected genes under positive selection when using the fSFS.

		uSFS		fSFS	
Population	Set	α	N	α	N
ZI	Analyzable	-0.032 ± (1.664)	7588	-0.032 ± (1.664)	5780
ZI	Negative	-4.698 ± (4.888)	339	-4.698 ± (4.888)	1690
ZI	Positive	0.775 ± (0.121)	2244	0.775 ± (0.121)	318
AFR	Analyzable	-0.679 ± (2.21)	3230	-0.679 ± (2.21)	1756
AFR	Negative	-5.375 ± (4.676)	244	-5.375 ± (4.676)	115
AFR	Positive	0.759 ± (0.121)	205	0.759 ± (0.121)	140
